# Quantifying Oxygen Management and Temperature and Light Dependencies of Nitrogen Fixation by Crocosphaera watsonii

**DOI:** 10.1128/mSphere.00531-19

**Published:** 2019-12-11

**Authors:** Keisuke Inomura, Curtis Deutsch, Samuel T. Wilson, Takako Masuda, Evelyn Lawrenz, Bučinská Lenka, Roman Sobotka, Julia M. Gauglitz, Mak A. Saito, Ondřej Prášil, Michael J. Follows

**Affiliations:** a School of Oceanography, University of Washington, Seattle, Washington, USA; b Daniel K. Inouye Center for Microbial Oceanography: Research and Education (C-MORE), University of Hawaii, Honolulu, Hawaii, USA; c Institute of Microbiology, The Czech Academy of Sciences, Třeboň, Czech Republic; d Collaborative Mass Spectrometry Innovation Center, Skaggs School of Pharmacy and Pharmaceutical Sciences, University of California, San Diego, San Diego, California, USA; e Marine Chemistry and Geochemistry Department and Biology Department, Woods Hole Oceanographic Institution, Woods Hole, Massachusetts, USA; f Department of Earth, Atmospheric and Planetary Sciences, Massachusetts Institute of Technology, Cambridge, Massachusetts, USA; University of Wyoming

**Keywords:** *Crocosphaera*, carbon, cell flux model, daily cycle, iron, light, nitrogen, nitrogen fixation, oxygen, photosynthesis, temperature

## Abstract

*Crocosphaera* is a major dinitrogen (N_2_)-fixing microorganism, providing bioavailable nitrogen (N) to marine ecosystems. The N_2_-fixing enzyme nitrogenase is deactivated by oxygen (O_2_), which is abundant in marine environments. Using a cellular scale model of *Crocosphaera* sp. and laboratory data, we quantify the role of three O_2_ management strategies by *Crocosphaera* sp.: size adjustment, reduced O_2_ diffusivity, and respiratory protection. Our model predicts that *Crocosphaera* cells increase their size under high O_2_. Using transmission electron microscopy, we show that starch granules and thylakoid membranes are located near the cytoplasmic membranes, forming a barrier for O_2_. The model indicates a critical role for respiration in protecting the rate of N_2_ fixation. Moreover, the rise in respiration rates and the decline in ambient O_2_ with temperature strengthen this mechanism in warmer water, providing a physiological rationale for the observed niche of *Crocosphaera* at temperatures exceeding 20°C. Our new measurements of the sensitivity to light intensity show that the rate of N_2_ fixation reaches saturation at a lower light intensity (∼100 μmol m^−2^ s^−1^) than photosynthesis and that both are similarly inhibited by light intensities of >500 μmol m^−2^ s^−1^. This suggests an explanation for the maximum population of *Crocosphaera* occurring slightly below the ocean surface.

**IMPORTANCE** *Crocosphaera* is one of the major N_2_-fixing microorganisms in the open ocean. On a global scale, the process of N_2_ fixation is important in balancing the N budget, but the factors governing the rate of N_2_ fixation remain poorly resolved. Here, we combine a mechanistic model and both previous and present laboratory studies of *Crocosphaera* to quantify how chemical factors such as C, N, Fe, and O_2_ and physical factors such as temperature and light affect N_2_ fixation. Our study shows that *Crocosphaera* combines multiple mechanisms to reduce intracellular O_2_ to protect the O_2_-sensitive N_2_-fixing enzyme. Our model, however, indicates that these protections are insufficient at low temperature due to reduced respiration and the rate of N_2_ fixation becomes severely limited. This provides a physiological explanation for why the geographic distribution of *Crocosphaera* is confined to the warm low-latitude ocean.

## Abstract (precis)

*Crocosphaera* is one of the major N_2_-fixing microorganisms in the open ocean. On a global scale, the process of N_2_ fixation is important in balancing the N budget, but the factors governing the rate of N_2_ fixation remain poorly resolved. Here, we combine a mechanistic model and both previous and present laboratory studies of *Crocosphaera* to quantify how chemical factors such as C, N, Fe, and O_2_ and physical factors such as temperature and light affect N_2_ fixation. Our study shows that *Crocosphaera* combines multiple mechanisms to reduce intracellular O_2_ to protect the O_2_-sensitive N_2_-fixing enzyme. Our model, however, indicates that these protections are insufficient at low temperature due to reduced respiration and the rate of N_2_ fixation becomes severely limited. This provides a physiological explanation for why the geographic distribution of *Crocosphaera* is confined to the warm low-latitude ocean.

## INTRODUCTION

Nitrogen (N) availability is recognized as a growth-limiting factor for primary producers in the ocean (1–4), controlling the flow of carbon (C) through the ecosystem (5–7). Dinitrogen fixation (N_2_ fixation) represents an important source of N that is estimated to account for nearly 50% of the fixed N input in the ocean (7). The microorganisms that fix N_2_ are termed “diazotrophs” and are physiologically diverse, including unicellular, filamentous, and heterocystous cyanobacteria with life strategies that include symbiotic, free-living, and colonial forms (8). Crocosphaera watsonii is a major phototrophic diazotroph and makes a significant contribution to the pool of fixed N in oligotrophic environments of the subtropical and tropical Atlantic and Pacific oceans (9–12). Observations show that the niche of *Crocosphaera* is limited to the region above 20°C (12, 13). Similarly, laboratory culturing experiments show that *Crocosphaera* grow only above 20°C (14, 15).

The enzyme responsible for N_2_ fixation, termed “nitrogenase,” is highly sensitive to oxygen (O_2_) (16, 17), thus necessitating careful O_2_ management by diazotrophs (18). In particular, *Crocosphaera* is a cyanobacterium with oxygen-producing photosynthesis, and fixing both C and N_2_ simultaneously would be a challenge. To circumvent the potential problem caused by O_2_ production, *Crocosphaera* fix N_2_ predominantly during the dark period in both laboratory (19–21) and natural (22) populations, thereby temporally segregating N_2_ fixation from O_2_-producing photosynthesis. However, O_2_ in the ocean is mostly saturated (∼200 μM in low latitudes) (23), and diurnal fluctuation of O_2_ is relatively small (∼10 μM) (24). Thus, even during the dark period, cells are likely to be exposed to a considerable influx of O_2_. Even at 5% O_2_ (∼50 μM), nitrogenase activity decreases to less than 30% within 20 min (17). Thus, questions remain about how *Crocosphaera* protects nitrogenase against O_2_.

O_2_ management by *Crocosphaera* must be considered within the context of their distinct daily physiological cycle. One metabolic strategy to constrain O_2_ is to sustain high rates of respiration in excess of the energetic demand during the night by using C stored from photosynthesis during the day (25). This distinct physiological cycle is paralleled by management of intracellular iron (Fe). *Crocosphaera* shuttles intracellular Fe between photosystems and nitrogenase during the daytime and the dark period to support photosynthesis and N_2_ fixation, respectively. The nitrogenase complex in particular is completely degraded and resynthesized each day (21). Despite the significance of the intracellular Fe cycling, the quantitative models have not mechanistically included the cycling nor have they linked it with other metabolisms, i.e., those affecting C, N, and O_2_. Developing a model which explicitly links Fe cycling to cellular metabolisms allows us to test how O_2_ can be managed within the context of the distinct daily cycle of Fe, C, N, and O_2_.

## 

### Physiological model of *Crocosphaera.*

First, we describe a new model of the C, N, Fe, and O_2_ budgets of *Crocosphaera*, and we then use it as a tool to explore the role of different O_2_ protection mechanisms. A more detailed description of the model is found in Materials and Methods and in Text S1 in the supplemental material.

10.1128/mSphere.00531-19.1 TEXT S1 Supplemental methods. Download Text S1, PDF file, 0.3 MB. Copyright © 2019 Inomura et al. 2019 Inomura et al. This content is distributed under the terms of the Creative Commons Attribution 4.0 International license.

### Simulating the daily cycle of metabolism based on Fe translocation.

**FIG 1:**
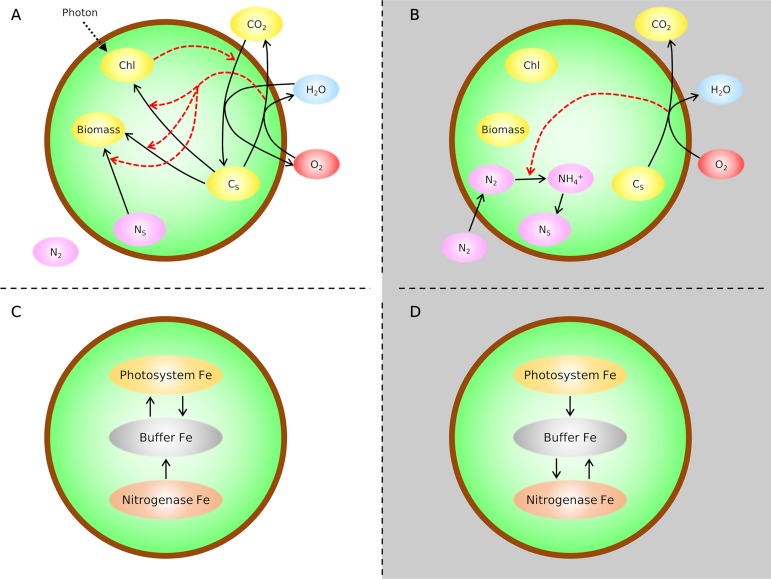
Schematics of modeled C, N, O_2_, and Fe fluxes. (A and B) C, N, and O_2_ fluxes for light and dark periods, respectively. Black solid arrows represent material fluxes, red dashed arrows represent energy fluxes, and a black dotted arrow represents photon flux. C-based molecules are in yellow, N-based molecules are in pink, O_2_ is in red, and H_2_O is in blue. C_S_ and N_S_, C and N storage, respectively; Chl, chlorophyll. (C and D) Fe fluxes (black solid arrows) for light and dark periods, respectively. The large circular frame indicates the cellular boundary.

We quantify the daily metabolic cycle for *Crocosphaera* by developing a coarse-grained model of *Crocosphaera* (cell flux model of *Crocosphaera* [CFM-Croco]), as depicted in Fig. 1 (see Materials and Methods and Text S1 for details). A previous dynamic model of *Crocosphaera* was developed to examine the daily cycle of C and N in *Crocosphaera* driven by a cellular clock and time-dependent functions (26). In our model, we explicitly link Fe cycles to C, N, and O_2_ metabolisms. As a starting point, we assume the total Fe constant within the cell and simulate the temporal variation of the fraction of Fe in different pools. During the light period, a predominant amount of Fe exists in photosystems contributing to photosynthesis. After sunset, Fe moves to nitrogenase, increasing the rate of N_2_ fixation. Before sunrise, Fe moves back to photosystems, preparing for daytime photosynthesis. We linearly link the amount of Fe in photosystems with the rate of photosynthesis and that in nitrogenase with the rate of N_2_ fixation leading to diurnal fluctuation of these metabolic rates. During the daytime, with photosynthesis, C storage (starch) is accumulated and fuels nitrogen fixation during the night. Nighttime metabolism also includes respiratory depletion of intracellular O_2_, which depends on the amount of C storage and the temperature-dependent metabolic capacity.

### Quantifying the rate of N_2_ fixation.

**FIG 2:**
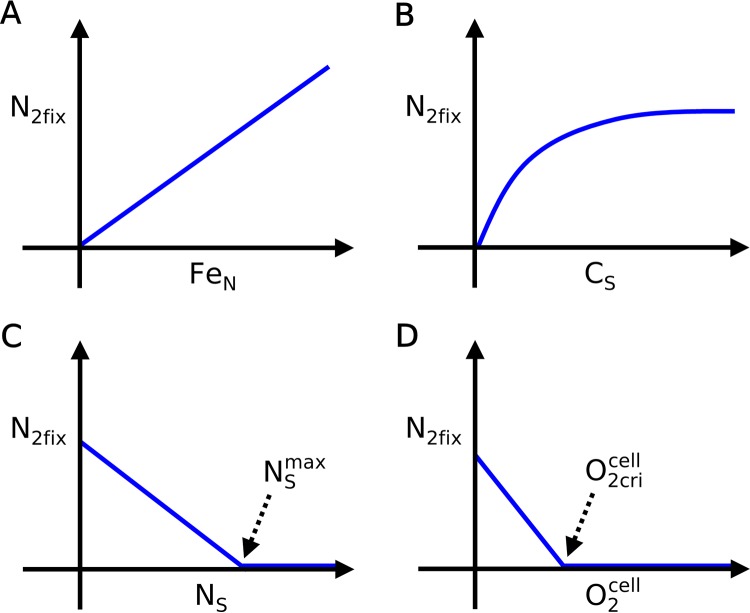
Schematics of how each factor influences N_2fix_ in the model. (A) Fe_N_; (B) C_S_; (C) N_S_; (D) $O_{\text{2}}^{\mathrm{cell}}$. $N_{\text{S}}^{\max}$ is the maximum N storage capacity, and $O_{\text{2cri}}^{\mathrm{cell}}$ is the critical O_2_ concentration.

N_2_ fixation is modeled as explicitly dependent on the intracellular concentration of nitrogenase, the size of the intracellular carbohydrate and fixed-N stores, the intracellular O_2_ concentration, and the temperature. During the dark period at a fixed temperature, the rate of N_2_ fixation (N_2fix_; mol N cell^−1^ h^−1^) is assumed to be affected by the fluctuation of Fe, the respiratory depletion of intracellular O_2_, and the storage of N and C as depicted in Fig. 2

(1) $$N_{\text{2fix}}=A_{\text{N2fix}}{\mathrm{Fe}}_{N}f_{N}\left(C_{S},N_{S},{\text{O}}_{2}^{\text{cell}}\right)$$

where *A*_N2fix_ is a rate constant (mol N mol Fe^−1^ h^−1^), Fe_N_ is the mass of Fe in nitrogenase (mol Fe cell^−1^), and *f*_N_(C_S_, N_S_, $O_{2}^{\text{cell}}$) scales the rate of N fixation between zero and its maximum value per nitrogenase (*A*_N2fix_), based on the available C storage (C_S_; mol C cell^−1^) and N storage (N_S_; mol N cell^−1^) and the presence of intracellular O_2_ ($O_{2}^{\text{cell}}$; mol O_2_ cell^−1^). Increasing nitrogenase concentrations (as proxied by Fe_N_) increase the encounter rate of N_2_ gas, with nitrogenase proportionally/linearly increasing N_2fix_ (Fig. 2A). C_S_ positively influences N_2fix_, since it provides energy for N_2_ fixation. We have assigned a saturating dependence (Michaelis-Menten type curve) to C_S_ (Fig. 2B), the C substrate. On the other hand, we assume that N_S_ negatively influences N_2_ fixation (Fig. 2C), as reactive N is often observed to inhibit N_2_ fixation (27–29). Intracellular O_2_ ($O_{2}^{\text{cell}}$) also negatively influences N_2_ fixation, since the proteins in the nitrogenase complex are sensitive to O_2_ (16, 17) (Fig. 2D). We assign a critical O_2_ concentration ($O_{\text{2cri}}^{\mathrm{cell}}$) above which N_2_ fixation does not occur, below which we assumed that N_2fix_ increases linearly with decreasing $O_{2}^{\text{cell}}$. This assumption is to represent an *in vitro* experiment of nitrogenase activities, where there are rather gradual negative correlations between O_2_ and activities of nitrogenase subunits (17).

### Simulating O_2_ management.

To simulate the intracellular O_2_ concentration, we assume a balance between diffusive flux into the cell and the respiratory O_2_ consumption within it, which can be expressed as follows (18):

(2) $$\left[O_{2}^{\text{cell}}\right]=\left[O_{2}^{}\right]-\frac{r^{2}\gamma_{\text{net}}}{3\kappa_{O_{2}}}$$

where $\left[O_{2}^{\text{cell}}\right]$ (mol O_2_ m^−3^) represents the intracellular concentration of O_2_, [O_2_] (mol O_2_ m^−3^) is the environmental concentration of O_2_, *r* is the cell radius (m), *γ*_net_ is the net respiration rate (respiration rate − photosynthesis) per cell volume (mol O_2_ m^−3^ s^−1^), and κ_O2_ is the effective O_2_ diffusion coefficient (m^2^ s^−1^) which accounts for the diffusivity in both the molecular boundary layer surrounding the cell and a semipermeable cell membrane layers. In order to minimize $\;\left[O_{2}^{\text{cell}}\right]$, cells may live in low [O_2_] environments, increase cell size (increasing *r*), increase respiration (increasing *γ*_net_), or decrease O_2_ diffusivity through the cell membrane layers (decreasing κ_O2_).

### Temperature dependence of metabolic processes.

To study why *Crocosphaera*’s niche exists mostly above 20°C, we use a commonly used temperature factor [*f_T_*(*T*)] based on the Arrhenius equation (30, 31):

(3) $$f_{T}\left(T\right)=\text{exp}\left(A_{T}\left(\frac{1}{T_{\text{ref}}}-\frac{1}{T}\right)\right)$$

where *T* is temperature (K), *T*_ref_ is a reference temperature (K), and *A_T_* is a constant factor (K^−1^). This factor independently modulates three key metabolic functions, namely, N_2_ fixation, photosynthesis, and respiration, and simulates the daily integrated rates of metabolisms. We explore the significance of the temperature dependence of each metabolic component.

### Quantifying light dependence of metabolisms with laboratory measurements.

We model the dependence of photosynthesis on light using the commonly employed saturating functional form (32, 33) with photoinhibition:

(4) $$f_{I}\left(I\right)=1-e^{A_{I}I}-\Omega \left(I\right)$$

where *I* is light intensity (μmol m^−2^ s^−1^), *A_I_* is a light absorption/processing factor (μmol^−1^ m^2^ s), and Ω(*I*) is a photoinhibition term (dimensionless). We have also conducted laboratory measurements of N_2_ fixation rates and photosynthetic electron transfer rates by Crocosphaera watsonii WH8501 for various light intensities (see Materials and Methods) and compared them with the model.

## RESULTS AND DISCUSSION

### Analysis of daily metabolic cycles.

**FIG 3:**
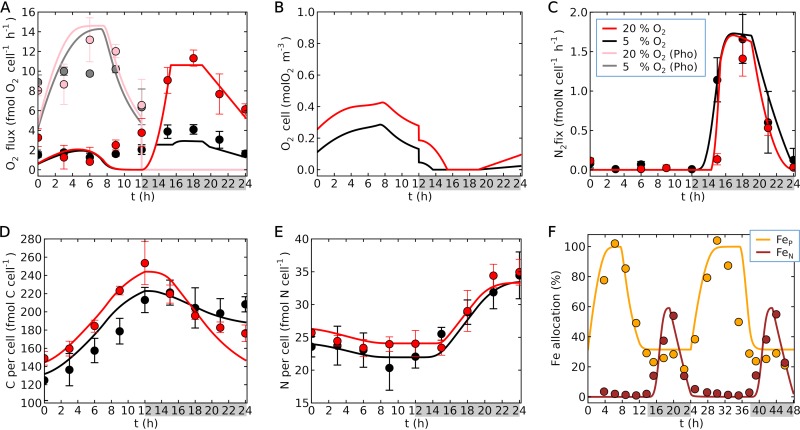
Simulated diurnal cycle of *Crocosphaera* for different O_2_ concentrations (curves) and laboratory data (circles). (A) O_2_ fluxes based on photosynthesis (gray and pink) and respiration (red and black). Here, O_2_ production is positive for photosynthesis, and O_2_ consumption is positive for respiration. (B) O_2_ concentrations in the cell. (C) N_2_ fixation rates. (D) C per cell. (E) N per cell. (F) Fe allocation. In panels A to E, error bars are the standard deviations of laboratory data (25). Here, we used a theoretical factor of 3 for C_2_H_4_:N_2_ (59). Red (or pink in panel A) is at 20% O_2_, and black (or gray in panel A) is at 5% O_2_. In panel F, Fe used by metabolism is based on quantitative protein data (21) for Fe in photosystems (Fe_P_; orange) and nitrogenase (Fe_N_; brown). In the model, the rest of the Fe exists in the buffer (an intracellular Fe storage). The key shown in panel C applies to panels A to E (pink and gray apply only to the photosynthesis (Pho) in panel A). In all panels, gray shading indicates dark periods. Temperature and light intensity are 28°C and 150 μmol m^−2^ s^−1^, respectively (21, 25).

We have simulated time-dependent laboratory cultures of *Crocosphaera* (25) and the linked Fe allocation within the cell (21). The model accurately predicted rates of photosynthesis and respiration both qualitatively and quantitatively for different O_2_ concentrations in the culture (Fig. 3A): 20% (186 μM, the normal atmospheric composition and surface ocean concentration in tropics) and 5% (46 μM, one-quarter of the normal composition), as used in the previous laboratory experiment (25). The rate of photosynthesis was maximal during the middle of the day for both 20% and 5% O_2_ values.

The rate of photosynthesis was correlated with Fe cycles, since the rate is proportional to Fe in photosystems (Fe_P_) (Fig. 3F); as the sun rises, Fe moves from the buffer (an intracellular Fe storage, e.g., ferritin and bacterioferritin [21]) to the photosystems, but in the afternoon, it starts moving back to the buffer, which was predicted by relating Fe to C_S_. As C_S_ increases and approaches maximum storage levels, there is no benefit to further photosynthesis. Therefore, the amount of photosystems was downgraded, and Fe was moved back to the buffer. We predicted a limited difference in photosynthesis between 5% and 20% O_2_, consistent with laboratory data.

We also reproduced the observed daily cycle of respiration and showed that higher respiration rates occurred at 20% O_2_ during the dark period due to respiratory protection (25). Because of this respiratory protection, intracellular O_2_ concentrations decreased to almost zero during the middle of the night (Fig. 3B), leading to peak N_2_ fixation during this period (Fig. 3C).

The data showed that N_2_ fixation increased more quickly at 5% O_2_ than at 20% O_2_ during the early dark period and can be explained by the concentration of intracellular O_2_. Based on the model, cellular O_2_ was eliminated more quickly at 5% (Fig. 3B), reaching zero before 14 h, while such elimination occurs after 15 h for 20% O_2_, thereby delaying N_2_ fixation. This delay in N_2_ fixation under normal O_2_ conditions is widely observed both in the laboratory (19, 34) and in natural populations (22). This delay may reflect the extra time required for O_2_ elimination, given that *Crocosphaera*’s *nifH* gene is transcribed following the initiation of the dark period (19, 21, 22), and both the model and laboratory data showed a much smaller delay in N_2_ fixation in a low-O_2_ environment. We capture this trend with the O_2_ dependence of N_2_ fixation (equation 1) (Fig. 2D); at 5% O_2_, the intracellular O_2_ is depleted quickly (Fig. 3B) and thus the rate of N_2_ fixation increases earlier (Fig. 3C).

During the dark period, as the nitrogenase enzyme is synthesized, Fe moves from the buffer to nitrogenase, initiating N_2_ fixation. As the dark period approaches dawn, Fe begins moving back to the buffer, preparing for daytime photosynthesis through degradation of the nitrogenase protein complex (19, 21), accompanied by decreased respiration (Fig. 3A), leading to the drop in the rate of N_2_ fixation (Fig. 3C) and increased intracellular O_2_ (Fig. 3B). We can use the model to consider how *Crocosphaera*’s diel cycle might be regulated. If the C storage reservoir were to be the trigger for Fe transfer, we would expect C storage to be significantly reduced during the dark period. However, even at the end of the dark period, we predict a significant amount of C storage remaining in the cell. If N storage is the trigger, Fe should start moving earlier in the case of 5% O_2_ than in the case of 20% O_2_, as the cell fixes N_2_ faster under low O_2_. The data show, however, that the rate of N_2_ fixation drops at almost the same time for the two O_2_ cases. Also, the peak of N_2_ fixation appears during similar time ranges among different studies (19, 21, 34). Finally, since *Crocosphaera* maintains the daily cycle even under continuous light (35, 36), it seems that the Fe transfer within *Crocosphaera* is largely controlled by a circadian clock that regulates key cellular functions. To further examine what controls Fe transfer, higher-resolution measurement of N_2_ fixation (34) under various O_2_ concentrations would also be useful.

Based on the metabolic rates (respiration, photosynthesis, cellular growth, and N_2_ fixation), we computed the cellular C and N quotas (Fig. 3D and E). During the light period, the cells accumulate C, while during the dark period, C storage decreases due to respiration and N_2_ fixation. During the light period, the data show that C accumulation is slightly greater for 20% O_2_ than for 5% O_2_; the model predicts this trend, with increased cell size for higher O_2_ (thus, larger cell size for 20% O_2_). During the dark period, however, it gets lower for 20% O_2_ than for 5% O_2_ , due to higher respiration for O_2_ management. Due to the larger cell size (discussed in “Sensitivity studies”), cells under 20% O_2_ have higher N. However, at the end of the dark period, cellular N levels under these different O_2_ concentrations get closer since the rate of N_2_ fixation is higher for 5% O_2_. The slight decrease in N during the early light period is due to cell division.

### Sensitivity studies.

In the following three sections, we describe sensitivity studies performed with the model to probe the significance of different O_2_ protection strategies. To examine the effect of each strategy, we specifically turned off each O_2_ management mechanism (size change, respiratory protection, and diffusion management) and the model results (cellular C, N, O_2_ fluxes, and N_2_ fixation rates) were compared with the default run with all the mechanisms present.

### Relationship between cell size and O_2_.

**FIG 4:**
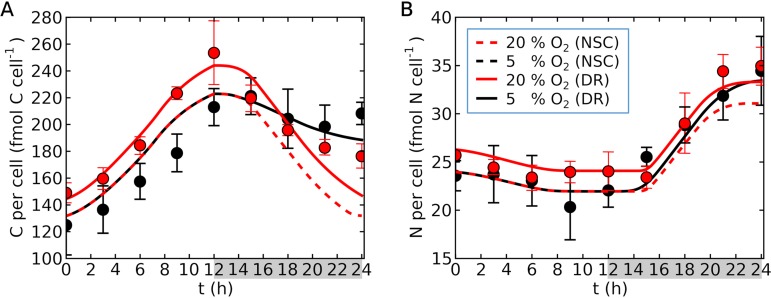
Simulated daily cycle of *Crocosphaera* with no size change based on O_2_ concentration. (A) C per cell. (B) N per cell. Curves represent simulations, and points with error bars (standard deviations) represent laboratory data (25). Here, we used a theoretical factor of 3 for C_2_H_4_:N_2_ (59). Dashed curves represent the run with no size change (NSC), and solid curves represent the default run (DR) (as in Fig. 3). Red indicates 20% O_2_, and black indicates 5% O_2_. NSC and DR show the same results for 5% O_2_ since the same size value is used. The key in panel B applies to both panels. Gray shading on the *x* axis indicates the dark period. Temperature and light intensity are 28°C and 150 μmol m^−2^ s^−1^, respectively (25).

An increase in cell size is a potential physiological strategy for *Crocosphaera*, since it decreases the surface-to-volume ratio, thereby decreasing passive O_2_ uptake per volume (equation 2) (18). Adjusting the cell size based on O_2_ concentrations facilitates replication of the laboratory data (Fig. 4).

During the light period, if the cell size is independent from O_2_ concentration, the model shows identical values for C and N per cell for different O_2_ concentrations, while the data show generally higher values for 20% O_2_; as a result, more predictions are outside the error bars of the data, especially for C per cell (Fig. 4A). During the dark period, the data show a reverse effect in relation to C: the C per cell in 20% O_2_ starts at a higher value but ends up at a lower value. This trend was reproduced only by including the size variation (Fig. 4A). N per cell is almost the same value at the end of the dark period, but the model with a fixed cell size shows much lower values in 20% O_2_ outside the error bar (Fig. 4B). These results indicate that the cells acclimate to higher O_2_ environments by adjusting their size. Recent studies have shown that there are two size classes of *Crocosphaera* (14, 22, 37). The model indicates that the larger cells have a significant advantage in O_2_ management and might be a result of adapting to the high O_2_ environments widespread in oceanic surface waters.

### Respiratory protection against O_2_.

**FIG 5:**
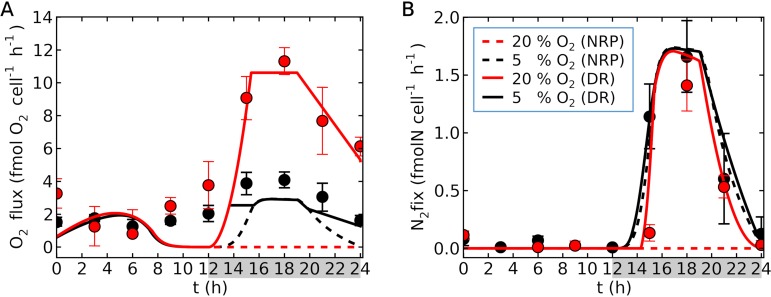
Simulated daily cycle of *Crocosphaera* with no respiratory protection. (A) Respiration. (B) N_2_ fixation. Curves represent simulations, and points with error bars (standard deviation) represent laboratory data (25). Here, we used a theoretical factor of 3 for C_2_H_4_:N_2_ (59). Dashed curves represent the run with no respiratory protection (NRP), and solid curves represent the default run (DR) (as in Fig. 3). Red indicates 20% O_2_, and black indicates 5% O_2_. The key in panel B applies to both panels. Gray shading on the *x* axis indicates the dark period. Temperature and light intensity are 28°C and 150 μmol m^−2^ s^−1^, respectively (25).

In order to examine whether respiratory protection is essential, we ran the model without respiratory protection (Fig. 5; see Fig. S1 in the supplemental material). In this case, respiration serves only to provide energy for N_2_ fixation. At 5% O_2_, the N_2_ fixation rate is almost the same as that for the simulation with respiratory protection (Fig. 5B). Once the dark period initiates and photosynthesis stops, the cellular O_2_ concentration drops low enough for N_2_ fixation. As N_2_ fixation initiates, respiration increases to provide energy, further decreasing intracellular O_2_ to zero, until N_2_ fixation peaks. On the other hand, at 20% O_2_, even after the initiation of the dark period, the cellular O_2_ concentration was still high (Fig. S1A), preventing N_2_ fixation (Fig. 5B). The respiration rate was much lower than the laboratory values (Fig. 5A), especially at 20% O_2_. Since there is no flux that consumes C at 20%, the model overestimated intracellular C (Fig. S1B). Together these results imply that respiratory protection is occurring in *Crocosphaera* and is essential for N_2_ fixation at normal O_2_ concentrations in the environment.

10.1128/mSphere.00531-19.2 FIG S1 Simulated daily cycle of *Crocosphaera* with no respiratory protection. (A) O_2_ concentration in the cell; (B) C per cell. Curves represent simulations, and points with error bars (standard deviations) represent laboratory data (25). Here, we used a theoretical factor of 3 for C_2_H_4_:N_2_ (59). Dashed curves represent the run with no respiratory protection (NRP), and solid curves represent the default run (DR) (as in Fig. 3). Red represents 20% O_2_, and black represents 5% O_2_. The key in panel A applies to both panels. Gray shading on the *x* axis indicates the dark period. Temperature and light intensity are 28°C and 150 μmol m^−2^ s^−1^, respectively (25). Download FIG S1, TIF file, 0.4 MB. Copyright © 2019 Inomura et al. 2019 Inomura et al. This content is distributed under the terms of the Creative Commons Attribution 4.0 International license.

### Diffusion management.

A model-data comparison indicates that the diffusivity of the cell membrane layers must be extremely low relative to both the diffusivity in the molecular boundary layer and to the diffusivity of the cell membrane layers inferred from other N_2_-fixing organisms. To achieve the results illustrated in Fig. 3, the effective diffusivity of O_2_ across the cell membrane layers must be set to 1/(6.45 × 10^4^) of the diffusivity of O_2_ in water. Previous studies of the heterotrophic and photoautotrophic diazotrophs Azotobacter vinelandii and *Trichodesmium* also inferred low cell wall permeability [1/(1.27 × 10^3^) (18, 38) and 1/(1.60 × 10^3^) (39) of the diffusivity of O_2_ in water, respectively], but the above predicted value for *Crocosphaera* is even lower. In contrast, the permeability of non-N_2_-fixing bacterial cells is much higher [1/(5.30 × 10^2^)] (40). Applying the value inferred for *Azotobacter* results in poor simulations of the laboratory data (Fig. S2), significantly overestimating the respiration at 5% O_2_ (Fig. S2A) and suppressing N_2_ fixation (Fig. S2B) because the O_2_ influx could not be matched. We conclude that *Crocosphaera* and other N_2_-fixing microbes necessarily control the cell wall permeability for O_2_; otherwise, N_2_ fixation would be impossible. However, the required protection varies between species, and the details of this adjustment need to be further investigated.

10.1128/mSphere.00531-19.3 FIG S2 Simulation of daily cycle of *Crocosphaera* with higher (rather normal) diffusivity of cell membrane layers. (A) Respiration; (B) N_2_ fixation; (C) O_2_ concentration in the cell; (D) C per cell. Curves represent simulations, and points with error bars (standard deviations) represent laboratory data (25). Here, we used a theoretical factor of 3 for C_2_H_4_:N_2_ (59). Dashed curves represent the runs with higher (rather normal) diffusivity (HD) of the cell membrane layers, and solid curves represent the default run (DR) (as in Fig. 3). Red represents 20% O_2_, and black represents 5% O_2_. The key in panel B applies to all the panels. Gray shading on the *x* axis indicates the dark period. Temperature and light intensity are 28°C and 150 μmol m^−2^ s^−1^, respectively (25). Download FIG S2, TIF file, 0.7 MB. Copyright © 2019 Inomura et al. 2019 Inomura et al. This content is distributed under the terms of the Creative Commons Attribution 4.0 International license.

**FIG 6:**
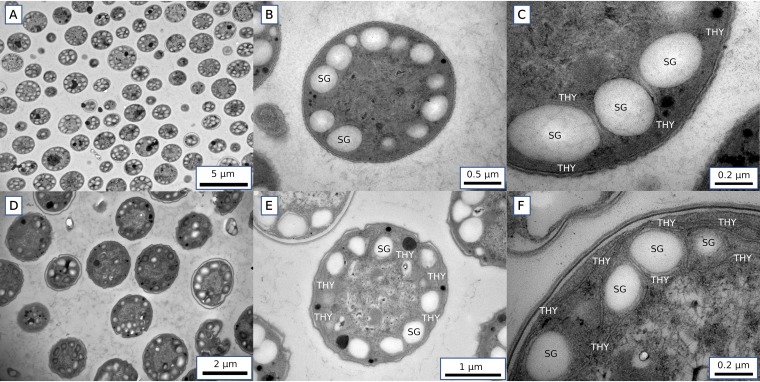
Transmission electron micrographs of *Crocosphaera* cells harvested at the 6-h time point during the light period (A to C) and at the 6-h time point during the dark (D to F). Starch granules (SG) and thylakoid membranes (THY) are observed mostly on the edge of the cytosol. More-detailed images (C and F) show that SG are observed mostly between THY.

One possibility is that carbohydrate storage may act as an O_2_ barrier. As shown previously (41), during cell division, starch granules are accumulated near the cell membranes rather than spread evenly in the cytoplasm. Our original electron microscopy images of *Crocosphaera* ultrathin sections revealed that the location of the granules close to the membranes is well preserved in both the light and dark periods (Fig. 6). Since the starch granules are relatively rigid and have dense hydrophilic structures, it is likely that they act as a barrier against O_2_ during the night. The images also show thylakoid membranes surrounding the granules (Fig. 6) (see also reference 41). Since respiration occurs on the thylakoid membranes as well as cellular membranes (42), such localization may make it possible for the cells to consume O_2_ before it reaches the inner cytoplasm, as well as further physically decreasing the diffusivity of O_2_.

Another possible barrier against O_2_ diffusion is the production of extracellular polymeric substances (EPS), which may create a thick hydrophilic layer, where the diffusion of O_2_ molecules is reduced. It has been hypothesized that Azotobacter vinelandii excrete alginate (one kind of EPS) to decrease the passive O_2_ uptake and thus protect nitrogenase (43). Its effect on N_2_ fixation has been further studied using quantitative modeling (18) and laboratory studies (44), supporting the hypothesis. In a batch culture, *Crocosphaera* produce EPS roughly proportional to their growth (37) and the production of EPS increases during the dark period (41). Given these findings, we hypothesize that the EPS produced by *Crocosphaera* plays an important role in reducing O_2_ diffusion. Recent observations suggest that hopanoid lipids may also play a role in diffusion management, as they have biochemical properties that potentially decrease the permeability to extracellular O_2_ and hopanoid synthesis genes are specifically observed in non-heterocyst-forming cyanobacterial diazotrophs (45).

### Temperature dependence of N_2_ fixation.

As we illustrate below, the model suggests that the temperature dependence of N_2_ fixation, and thus the fitness of *Crocosphaera*, is largely explained by the temperature dependence of respiration. N_2_ fixation by *Crocosphaera* is observed to have a strong dependence on temperature. Cell-specific rates of N_2_ fixation are maximal at approximately 30°C and decrease to almost zero at 22°C (15). These laboratory-derived physiological observations are supported by field observations of *Crocosphaera* being most prevalent in warm oceanic regions above 20°C (12, 13).

**FIG 7:**
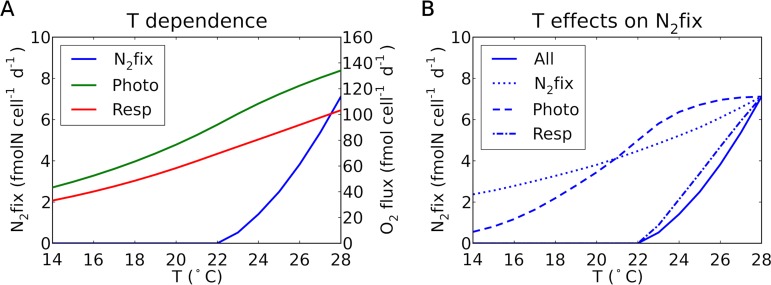
Simulated temperature dependence on N_2_ fixation, photosynthesis, and respiration. (A) Simulated temperature dependence on these metabolic fluxes when the temperature function is assigned to all of these fluxes. N_2_fix, N_2_ fixation; Resp, respiration; Photo, photosynthesis. (B) Impact of each temperature dependence on N_2_ fixation rate. The solid curve represents when all three fluxes are temperature dependent. The other curves represent when only one of these fluxes is temperature dependent (see the key in the figure; i.e., N_2_fix, Photo, and Resp indicate that the temperature function is assigned only to N_2_ fixation, photosynthesis, and respiration, respectively). A light intensity of 150 μmol m^−2^ s^−1^ is used (25). Also, a saturating O_2_ concentration is used based on the specified temperature and a salinity of 35 (60). The temperature dependence of diffusivity is given based on Walden’s rule (61) and the temperature-dependent viscosity of water (62). The diffusivity of the cell membrane layers is assumed to be proportional to that of water (18).

In the model, N_2_ fixation, photosynthesis, and respiration are each modeled with independent, Arrhenius equation-like temperature dependence (equation 3). By employing these in combination, the model replicates the observed temperature dependence of N_2_ fixation (Fig. 7A). We tested which temperature dependence has the strongest effect by applying equation 3 to only one of the metabolisms (Fig. 7B). The results show that the temperature dependence on respiration has the strongest effect, closely representing the predicted results with all the temperature dependences. This indicates that the negative effect of temperature on N_2_ fixation is largely due to decreased rates of respiration being insufficient to draw down intracellular O_2_, and therefore nitrogenase is unable to fix N_2_. A decrease in the rate of respiration will also decrease the supply of energy for nitrogenase. Decreased temperature will also have effects on other related metabolic processes, such as a direct effect on the rate of enzymatic activity of nitrogenase and a decrease in photosynthesis and therefore less C storage for respiration (Fig. 7B). All of these impacts, however, turned out to be smaller than the decrease in respiratory protection against O_2_.

### The rate of N_2_ fixation maximized at moderate light intensity.

**FIG 8:**
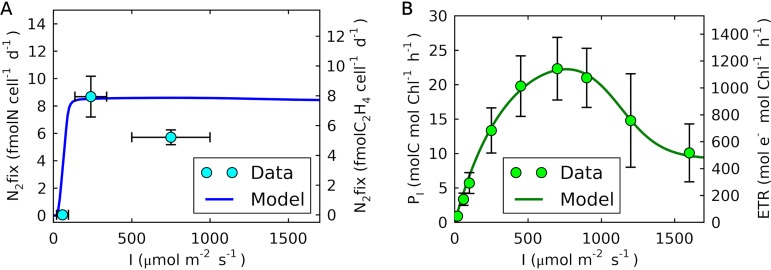
Experimental data and model simulation of the light dependence of daily integrated rates of N_2_ fixation and photosynthesis of *Crocosphaera*. (A) Simulated light dependence of the N_2_ fixation rate of *Crocosphaera* at 20% O_2_ (left axis) compared with the acetylene reduction data obtained in this study (right axis). (B) Simulated light dependence of the photosynthesis rate (left axis) compared with the average daytime ETR (electron transfer rate) measured in this study (right axis). Error bars represent the standard deviations of the samples. A temperature of 28°C was used.

To examine light dependence on N_2_ fixation for *Crocosphaera*, we have simulated the rate of N_2_ fixation at various light intensities. In the model, the light intensity influences the rate of photosynthesis based on the equation of light saturation and photoinhibition (equation 4). The model shows that the rate of N_2_ fixation increases at low light intensity due to increased photosynthesis and, thus, increased C storage (Fig. 8A). However, despite photosynthesis rates increasing with light intensities up to ∼700 μmol m^−2^ s^−1^ (Fig. 8B), N_2_ fixation saturates at a relatively low light intensity (∼100 μmol m^−2^ s^−1^) (Fig. 8A), since it becomes limited by the availability of nitrogenase (here proxied by Fe_N_). This prediction is confirmed by our original measurements of the rates of acetylene reduction (proxy for N_2_ fixation) and ETR (photosynthetic electron transfer rate) for various light intensities from this study. Above 140 μmol m^−2^ s^−1^, the rate of N_2_ fixation becomes stable despite the significant increase in ETR.

Our measurements also show that above certain light intensities, both of the rates (N_2_ fixation and photosynthesis) start dropping (Fig. 8), likely due to photoinhibition. These results may explain why the maximum population of *Crocosphaera* is often observed below the surface but above a depth of 50 m (12, 13), where they can receive moderate light intensities (100 to 400 μmol m^−2^ s^−1^), enough for N_2_ fixation without photoinhibition. The model resolves photoinhibition, capturing the trend in ETR (Fig. 8B). However, it does not predict the observed decrease in N_2_ fixation under strong light, despite the drop in photosynthesis above 700 μmol m^− 2^ s^−1^, meaning that the rate of photosynthesis or the level of C storage cannot explain the decreasing trend in N_2_ fixation (Fig. 8A). This indicates that high light intensity rather directly inhibits nitrogen fixation, potentially by damaging machinery for nitrogenase synthesis or subunits of nitrogenase before its assembly.

### Conclusions.

We have developed a coarse-grained model of *Crocosphaera* (CFM-Croco) and simulated a daily cycle of *Crocosphaera* metabolism with distinct C, N, O_2_, and Fe fluxes. The model mechanistically links the diurnal cycle of Fe with that of C, N, and O_2_, reproducing published observations (21, 25). This indicates that including the Fe cycle is essential for simulating the diurnal cycle of *Crocosphaera* metabolism and nutrient fluxes. The model results suggest that *Crocosphaera* employs multiple mechanisms to manage intracellular O_2_: size change, respiratory protection, and decreased diffusivity. Since respiratory O_2_ management is crucial for N_2_ fixation, the temperature dependence of respiration has a significant impact on N_2_ fixation, which provides a hypothesis for the strong temperature constraint on their growth and niche in the environment. The light dependence of photosynthesis does not give an advantage to N_2_ fixation under extremely high light due to photoinhibition and limitations on nitrogenase concentration, the latter of which may be constrained by the intracellular space. This indicates that the optimum depth is likely not at the very surface, despite the potentially highest availability of Fe dust.

## MATERIALS AND METHODS

### Cell flux model of *Crocosphaera*.

Here, we describe the algorithms employed to implement the model. The model schematic is depicted in Fig. 1. Time-dependent equations are given to describe the rates of change in each of the macromolecular pools (Table 1). The fluxes between macromolecular pools are quantified at each time step. The time-dependent equations (Tables 1 and 2) are advanced in finite time steps, updating the status of the cells. Parameter values and nomenclature with units are available in Tables S1 and S2 in the supplemental material, respectively.

**TABLE 1:** Fundamental relations of C-, N-, and O_2_-based molecules

Equation^*a*^	Equation no.
$\;\frac{dC_{S}^{}}{dt}=P_{I}^{}\text{Chl}-\lambda \left(1+E\right)-P_{\mathrm{CO2}}^{\mathrm{N2fix}}-P_{\mathrm{CO2}}^{\mathrm{RP}}-Exc$	5
$\frac{\mathrm{dX}}{\mathrm{dt}}=\frac{X\lambda }{Q_{C}^{}}$	6
$\;\frac{dN_{S}}{dt}=N_{\text{2fix}}-\lambda Y_{\text{bio-all}}^{\text{N:C}}$	7
$\frac{d{\text{O}}_{2}^{}}{\mathrm{dt}}=P_{\text{O2}}^{}-R_{\text{O2}}^{}+V_{\text{O2}}^{}$	8

a C_S_, C storage; *t*, time; *P_I_*, photosynthesis rate per chlorophyll; Chl, chlorophyll; λ, biomass production rate; *E*, conversion factor of biomass production to biosynthetic CO_2_ production; N_2fix_, N_2_ fixation rate; $P_{\text{CO2}}^{\text{N2fix}}$, CO_2_ production due to electron donation to and respiratory energy production for N_2_ fixation; $P_{\text{CO2}}^{\text{RP}}$, CO_2_ production due to respiratory protection; *Exc*, C excretion rate; *X*, population density of cells; *Q*_C_, cellular C quota; N_S_, N storage per cell;$\;Y_{\text{bio-all}}^{\text{N:C}}$, N:C of biomass including storage; O_2_, O_2_ per cell; *P*_O2_, O_2_ production rate; *R*_O2_, respiration rate; *V*_O2_; O_2_ exchange by diffusion.

**TABLE 2:** Fundamental relationships of Fe-related molecules

Equation^*a*^	Equation no.
$\frac{d{\text{Fe}}_{\text{P}}^{}}{dt}=F_{B}^{\text{P}}-F_{\text{P}}^{B}$	9
$\frac{d{\text{Fe}}_{B}^{}}{dt}=-F_{B}^{\text{P}}+F_{\text{P}}^{B}-F_{B}^{N}+F_{N}^{B}$	10
$\frac{d{\text{Fe}}_{N}^{}}{dt}=F_{B}^{N}-F_{N}^{B}$	11
$\frac{d\text{Chl}}{dt}=\left(F_{B}^{\text{P}}-F_{\text{P}}^{B}\right)Y_{\text{photo}}^{\text{Chl:Fe}}$	12

a Fe_P_, Fe in the photosystems; $F_{B}^{\text{P}}$ and $F_{\text{P}}^{B}$, translocation of Fe from the buffer to the photosystem and vice versa; Fe_B_, Fe in the buffer; $F_{B}^{N}$ and $F_{N}^{B}$ , translocation of Fe from the buffer to nitrogenase and vice versa; Fe_N_, Fe in nitrogenase;$\;Y_{\text{photo}}^{\text{Chl:Fe}}$, Chl:Fe in the photosystems.

10.1128/mSphere.00531-19.4 TABLE S1 Values used for tunable parameters. Download Table S1, PDF file, 0.2 MB. Copyright © 2019 Inomura et al. 2019 Inomura et al. This content is distributed under the terms of the Creative Commons Attribution 4.0 International license.

10.1128/mSphere.00531-19.5 TABLE S2 Parameters, definitions, and units used in both the main text and the supplemental material; the parameters are listed roughly in order of appearance from the main text to Text S1 (supplemental methods). Download Table S2, PDF file, 0.2 MB. Copyright © 2019 Inomura et al. 2019 Inomura et al. This content is distributed under the terms of the Creative Commons Attribution 4.0 International license.

### C, N, and O_2_ metabolism.

In order to compute the time variation of intracellular C, N, and O_2_ pools and the cell density, we consider the chemical fluxes that impact them. Specifically, we include time variation of C_S_, cell population density (*X*) based on biomass production, N_S_, and O_2_ (equations given in Table 1 and schematics given in Fig. 1A and B). The balance in C storage pool C_S_ (equation 5) is based on the balance among photosynthesis (*P_I_* Chl), biomass production (*λ*), biosynthetic CO_2_ production (*E*λ), CO_2_ production for N_2_ fixation ($P_{\text{CO2}}^{\text{N2fix}}$), CO_2_ production due to respiratory protection ($P_{\text{CO2}}^{\text{RP}}$), and C excretion (*Exc*). Cellular C and N are the sum of the baseline biomass and the C and N storages, respectively.

We assume that biomass production is used for the production of new cells, which drives the time change in population density (equation 6). Biomass production is supported not only by C but also by N. Thus, we consider the effect of biomass production on N_5_ ($\lambda Y_{\text{bio-all}}^{\text{N:C}}$) (equation 7), which is balanced by N_2_ fixation (N_2fix_). Finally, the O_2_ budget is based on photosynthesis (O_2_ production rate [*P*_O2_]), the respiration rate (*R*_O2_), and the diffusive exchange of O_2_ (*V*_O2_) (equation 8).

Since the metabolism differs between the light and dark periods, we employed different parameterizations of the fluxes in Table 1 at different times of day. Specifically, *P_I_*, *λ*, *Exc*, and *P*_O2_ are unique to the light period, and N_2fix_, $P_{\text{CO2}}^{\text{N2fix}}$, and $P_{\text{CO2}}^{\text{RP}}$ are specific to the dark period.

In order to solve the model equations, we have applied a finite-difference method to equations 5 to 7. Since the time scale of O_2_ concentration is small relative to that of other metabolites (C, N, Fe), we have assumed a pseudo-steady state; thus, O_2_ uptake and O_2_ production are always balanced by respiration. For the calculation of fluxes that influence the time variation of each pool, we consider the size of the elemental pools and O_2_ concentrations (the details of the flux calculations are described in Text S1 in the supplemental material).

### Fe metabolism.

The rates of C and N_2_ fixation both depend on the Fe allocation to the enzymes which mediate those processes. Here, we model the time-dependent allocation to those Fe pools. The time-dependent equations for the Fe system are given in Table 2. We assume that the exchange of Fe between photosystems and nitrogenase is mediated by an Fe buffer, such as bacterioferritin protein (Fig. 1C and D) (21). Thus, the time variation of Fe in the photosystems is based on its exchange with the buffer Fe pool (equation 9). The buffer Fe pool is influenced not only by the Fe from the photosystems but also by the exchange of Fe with nitrogenase (equation 10). The Fe allocation to nitrogenase results from the balance between the loss to and gain from the buffer (equation 11).

### Connecting Fe fluxes to C, N, and O_2_ fluxes.

The amount of Fe in photosystems (Fe_P_) proportionally influences photosynthesis, thus impacting C and O_2_ fluxes. We have assumed that the ratio of chlorophyll to Fe in a photosynthetic apparatus (mol C mol Fe^−1^) is constant ($Y_{\text{photo}}^{\text{Chl:Fe}}$); thus, the balance in chlorophyll is proportional to the balance in photosystem Fe (equation 12). The amount of chlorophyll in turn influences the rate of photosynthesis (equation 5). The rate of N_2_ fixation (N_2fix_) is assumed to be proportional to the amount of nitrogenase proxied by Fe in nitrogenase (Fe_N_). Thus, through the rate of N_2_ fixation, Fe_N_ influences C and N fluxes, and through the associated respiration providing energy for N_2_ fixation, it can influence O_2_ fluxes.

In order to calculate the amount of Fe pools and chlorophyll, we applied a finite-difference method to equations 9 to 12. For the computation of Fe fluxes, we considered various factors, such as the size of the Fe pool of the origin and the destination, time, O_2_ concentration, and carbohydrate storage. Fe fluxes are parameterized based on these factors to reproduce the laboratory observations (21) (Fig. 3F) (see Text S1 in the supplemental material).

### Differentiating light and dark periods.

To reflect a distinct diurnal cycle of *Crocosphaera*, we resolve differences in metabolic configuration during the day and night; some fluxes in Tables 1 and 2 are specific to a certain time of day. The schematics of which flux applies to each time period are illustrated in Fig. 1. The following section broadly describes the day-night differentiation. The detailed fluxes are described in Text S1.

### Light period.

During the light period, cells can harvest light and fix C, accumulating C storage and producing biomass (Fig. 1A). However, N_2_ fixation is small and accordingly respiratory protection is also small. For the N source for biomass production, the cell relies on N storage. To reflect this, all the terms in equations 5 to 8 (Table 1) are used except N_2fix_, $P_{\text{CO2}}^{\text{N2fix}}$, and $P_{\text{CO2}}^{\text{RP}}$ (∼0). Also, we assume that the translocation of Fe from the buffer to nitrogenase ($F_{B}^{N}$) is ∼0, since no Fe_N_ was observed during the light period (21); this assumption depletes Fe_N_ (Fig. 1C).

### Dark period.

During the dark period, photosynthesis does not occur, but the cell uses stored C for respiration and N_2_ fixation (Fig. 1B). Also, we assume that biomass production and excretion do not occur. Thus, in equation 5, *P_I_* = λ *= Exc *= 0. This assumption allows accumulating N storage with N_2_ fixation, as observed previously (25). According to the observed targeted proteomics (21), there is limited net Fe flux to the photosystems, and we assume that the translocation of Fe from the buffer to the photosystem ($F_{B}^{P}$) is zero. This assumption creates the flow of Fe from photosystems to nitrogenase (Fig. 1D), increasing the rate of N_2_ fixation during the early dark period. During the later dark period, we assume that $F_{B}^{N}$ is ∼0, and the model forces the movement of Fe from nitrogenase to buffer as predicted (21).

### Simulating temperature and light dependences on metabolisms.

The temperature dependence is simulated based on applying a temperature factor *f_T_*(*T*) (equation 3) to N_2_ fixation, photosynthesis, and respiration. To test the effect of temperature dependence on each metabolism, we applied *f_T_*(*T*) to only one of these metabolisms and plotted the rate of N_2_ fixation (Fig. 7B). To represent the light dependence of photosynthesis, we have applied a light factor, *f_I_*(*I*), (equation 4) to the maximum rate of photosynthesis.

### Preparing Crocosphaera watsonii WH8501.

Stock cultures of Crocosphaera watsonii WH8501 were obtained from the Culture Collection Yerseke (The Royal Netherlands Institute for Sea Research, Yerseke, The Netherlands; strain number CCY 0601). The cells were maintained in N-free YBC-II medium (46) at 28°C in glass flasks under constant white light of 150 μmol μm^−2^ s^−1^ using a 12-h:12-h light-dark (12L:12D) cycle. At the beginning of each experiment, the cultures were transferred into flat-panel photobioreactors (FMT150; Photon System Instruments, Brno, Czech Republic) (47) with a sinusoidal 12L:12D growth irradiance peaking at 400 μmol m^−2^ s^−1^ with aeration. Cultures were acclimated to these conditions and maintained in exponential growth for at least 5 generations (about 15 days).

### Transmission electron microscopy.

*Crocosphaera* cells (∼10^8^ cells ml^−1^) were harvested by centrifugation (5 min at 5,000 × *g*). The cells were resuspended in 1 volume of the growth medium mixed 1:1 with 5% (vol/vol) glutaraldehyde fixative in 0.2 M cacodylate buffer, pH 7.2. After 15 min of rotary shaking at room temperature, cells were transferred to 0.1 M cacodylate buffer containing 2.5% (vol/vol) glutaraldehyde and fixed overnight at 4°C. Pelleted cells were washed with cacodylate buffer and postfixed with 1% (wt/vol) osmium tetroxide for 2 h. After washing steps with the same buffer, cells were dehydrated through a graded series of acetone, embedded in low-viscosity Spurr resin (EMS), and polymerized at 60°C for 48 h. Ultrathin sections of 60 nm were cut using an ultramicrotome (UCT, Leica). Sections were collected on Formvar-coated copper grids and stained with 1% (wt/vol) aqueous uranyl acetate for 10 min and with Sato’s lead citrate for 3 min (48). Prepared sections were examined in a JEOL 1010 transmission electron microscope (JEOL) equipped with a Mega View III camera (SIS). Acquired pictures were analyzed by ImageJ software (49).

### N_2_ fixation measurements.

To determine the rates of N_2_ fixation by acetylene reduction assays (50), 5 ml of cell suspensions grown under different light intensities were dispensed into HCl-rinsed glass vials. After each vial was sealed with a septum, 10 ml of acetylene gas (99.7% [vol/vol]; Linde Gas) was injected by replacing the same volume of headspace. The samples were incubated at 28°C in the dark for 12 h. Subsamples of the headspace were taken immediately after acetylene addition and then at the end of the incubation to measure their ethylene content with a flame ionization gas chromatograph (HRGC 5300; Carlo Erba Instruments). Ethylene production during the incubation was analyzed, and produced ethylene was calculated according to Breitbarth et al. (51).

### Variable-fluorescence light response curves.

The diel changes in the dependence of photosynthesis on light intensity was assessed with photosynthesis versus irradiance (P versus E) curves using the electron transfer rate (ETR) through photosystem II as a photosynthesis proxy. Cells were harvested in 2-h intervals throughout the diel cycle (52). Samples were acclimated to the dark for 10 min and placed inside a FL3500 fast repetition rate (FRR) fluorometer (Photon Systems Instruments, Czech Republic) maintained at the same temperature as the stock cultures. A series of 100 simultaneous blue (463 nm) and amber (617 nm) flashes of 1-μs duration was applied to induce a single turnover of the reaction centers of photosystem 2 (RCII) at 10 different light intensities ranging from 0 to 1,600 μmol quanta m^−2^ s^−1^ (0 to 840 μmol quanta m^−2^ s^−1^ of blue light combined with 0 to 600 μmol quanta m^−2^ s^−1^ of amber light). The resulting fluorescence light curves were fitted to the model of Kolber et al. (53) to derive the maximum (*F_m_*′), operational (*F*′), and minimum (*F_o_*′) fluorescence values at given actinic light, the effective PSII absorption cross-section (σ_PSII_), and the connectivity between photosystems (*p*). These parameters were then used to calculate the ETR (54):

(13) $$\text{ETR}=\sigma_{\text{PSII}}\times n_{\text{PSII}}\times \frac{F_{q}\prime }{F_{v}\prime }\times {\text{Φ}}_{\text{RCII}}\times E$$

where *E* is the intensity of the actinic light, Φ_RCII_ is the quantum yield of photochemistry within RCII [taking constant values of 1 mol *e*^−^ (mol photons^−1^)], *F_q_*′ is variable fluorescence in the light (*F_m_*′ − *F*′), *F_v_*′ is the maximal variable fluorescence in the light (*F_v_*′ = *F_m_*′ − *F_o_*′), and *n*_PSII_ is the ratio of functional reaction centers of PSII to total chlorophyll *a* (55, 56).

The ETR values were then plotted versus irradiance and modeled after the reports of Eilers and Peeters (57) and Silsbe and Kromkamp (58) to derive the maximum ETR (ETR_max_), the initial slope of the P versus E curve, and the light saturation point of the ETR (*E_K_*).

### Data availability.

The model developed in this paper as well as the plotted data have been uploaded in Zenodo/GitHub and is freely available from https://zenodo.org/record/3265448.
