# Erratum for Inomura et al., “Quantifying Oxygen Management and Temperature and Light Dependencies of Nitrogen Fixation by Crocosphaera watsonii”

**DOI:** 10.1128/mSphere.00075-20

**Published:** 2020-02-05

**Authors:** Keisuke Inomura, Curtis Deutsch, Samuel T. Wilson, Takako Masuda, Evelyn Lawrenz, Lenka Bučinská, Roman Sobotka, Julia M. Gauglitz, Mak A. Saito, Ondřej Prášil, Michael J. Follows

**Affiliations:** a School of Oceanography, University of Washington, Seattle, Washington, USA; b Daniel K. Inouye Center for Microbial Oceanography: Research and Education (C-MORE), University of Hawaii, Honolulu, Hawaii, USA; c Institute of Microbiology, The Czech Academy of Sciences, Třeboň, Czech Republic; d Collaborative Mass Spectrometry Innovation Center, Skaggs School of Pharmacy and Pharmaceutical Sciences, University of California, San Diego, San Diego, California, USA; e Marine Chemistry and Geochemistry Department and Biology Department, Woods Hole Oceanographic Institution, Woods Hole, Massachusetts, USA; f Department of Earth, Atmospheric, and Planetary Sciences, Massachusetts Institute of Technology, Cambridge, Massachusetts, USA

## ERRATUM

Volume 4, no. 6, e00531-19, 2019, https://doi.org/10.1128/mSphere.00531-19. This article was published on 11 December 2019 with the sixth author’s name presented incorrectly. The byline has been updated in the current version, posted on 30 January 2020.

