# Supplementary material for: Quantifying Oxygen Management and Temperature and Light Dependencies of Nitrogen Fixation by Crocosphaera watsonii
Source: mSphere. 2019 Dec 11;4(6):e00531-19. doi: 10.1128/mSphere.00531-19 (PMC6908418; doi:10.1128/mSphere.00531-19)
Supplement: TEXT S1 [file mSphere.00531-19-s0001.pdf]

Here we describe the model with equations. Parameter values and nomenclature are in Table S1, S2, respectively. The model code is freely available in Zenodo/GitHub at https://zenodo.org/record/3265448 (doi: 10.5281/zenodo.3265448).

Computation of C, N and O_2 metabolism

Light period. The rate of photosynthesis depends on the abundance of chlorophyll ([eq. 5] in the main text), which is dynamic and related to Fe allocation ([eq. 12] in the main text). We also compute an "ideal" abundance of Chlorophyll; that would support sufficient C fixation to match the demands of N limited biosynthesis, based on the stored N. The biomass production rate which determines the ideal chlorophyll concentration (mol C cell^{-1} s^{-1}) is thus equal to the N limited biomass production rate, described as follows by using Michaelis-Menten form:

$$\lambda^{Chl-ideal} = \lambda^{max} \frac{N_S}{N_S \ + K_{Ns} Y_{bio-all}^{N:C} / Y_{bio}^{N:C}} \qquad \text{[eq. S1]}$$

where λ^{max} (mol C cell^{-1} s^{-1}) is the maximum biomass production rate, N (mol N cell^{-1}) is the N storage, K_{Ns} (mol N cell^{-1}) is the half-saturation constant of N storage for biomass production, and $Y_{bio-all}^{N:C}$ and $Y_{bio}^{N:C}$ (mol N mol C^{-1}) are the N:C ratio of biomass with and without the nutrient storage, respectively.

Photosynthesis must meet the demands for storage to support night time activity. We compute the rate of C storage production based on the current magnitude of C storage:

$$P_{Cs}^{Chl-ideal} = (C_S^{max} - C_S \ ) R_{Cs} \qquad \text{[eq. S2]}$$

where $P_{Cs}^{Chl-ideal}$ (mol C cell^{-1} s^{-1}) is the ideal C storage production rate, C_S^{max} (mol C cell^{-1}) is the maximum C storage, C_S (mol C cell^{-1}) is the C storage, and R_{Cs} (s^{-1}) is the rate constant for the C storage production. If the C store is not at maximum capacity, photosynthesis is required to provide a supply at a rate sufficient to keep up with the prescribed storage rate, R_{Cs}.

By plugging [eq. S1] and [eq. S2] into [eq. 5] ($\lambda^{Chl\text{-}ideal}$ into λ and $P_{Cs}^{Chl-ideal}$ into dC_S/dt) and assuming $Exc = N_{2fix} = P_{CO2}^{N2fix} = 0$ and solving the equation for *Chl*, we obtain the "ideal" chlorophyll concentration Chl^{ideal} (mol C cell^{-1}):

$$Chl^{ideal} = \frac{\lambda^{Chl-ideal}(1+E) + P_I^{Chl-ideal}}{P_I} \quad \text{[eq. S3]}$$

Here, E (dimensionless) is the ratio of the carbohydrate production rate to the biomass production rate for providing energy for biosynthesis (1–3), and P_I (s^{-1}) is the photosynthesis rate per chlorophyll. For obtaining P_I, we use a "target theory" based equation used in many other studies (4–7) with the addition of a sigmoidal photo-inhibition term $\Omega(I)$:

$$P_I = P_I^{max}\left(1 - e^{-A_I I} - \Omega(I)\right) \quad \text{[eq. S4]}$$

$$\Omega(I) = \frac{B_I P_I^{max}}{1 + exp(-C_I I + D_I)} \quad \text{[eq. S5]}$$

where P_I^{max} (s^{-1}) is the maximum photosynthesis rate per chlorophyll, and A_I, B_I, C_I and D_I are constant parameters.

Since the chlorophyll concentration is typically not at this ideal state, we parameterize the synthesis terms accordingly. When $Chl^{ideal} > Chl$ where *Chl* is the actual chlorophyll amount (mol C cell^{-1}), the growth is limited by the amount of chlorophyll and C storage and biomass synthesis are accordingly reduced from their ideal rates by a factor M_{Chl} (dimensionless):

$$M_{Chl} = \frac{Chl}{Chl^{ideal}}. \quad \text{[eq. S6]}$$

Alternatively, when $Chl^{ideal} \le Chl$,

$$M_{Chl} = 1. \quad \text{[eq. S7]}$$

Hence biomass production rate λ (mol C cell^{-1} s^{-1}) is evaluated as follows:

$$\lambda = \lambda^{Chl-ideal} M_{Chl}. \qquad \text{[eq. S8]}$$

When $Chl_{ideal} < Chl$, the extra C fixed that exceeds the potential rate of C storage production is assumed to be excreted to the environment at a rate given by:

$$Exc = P_I \ Chl - \lambda(1 + E) - (C_S^{max} - C_S \ )R_{Cs} \qquad \text{[eq. S9]}$$

but otherwise, $Exc = 0$. Once biomass production rate λ is computed, we can energetically and stoichiometrically relate λ to the biomass production balanced respiration rate R_{Bio} (mol O_2 cell^{-1} s^{-1}) (1–3), and to consumption of the N storage dN_S/dt (mol N cell^{-1} s^{-1}) respectively:

$$R_{Bio} = \lambda Y_{synth}^{O2:bio} \qquad \text{[eq. S10]}$$

and from [eq. 7],

$$\frac{dN_S}{dt} = -\lambda Y_{bio-all}^{N:C} \qquad \text{[eq. S11]}$$

where $Y_{synth}^{O2:bio}$ (mol O_2 mol C^{-1}) is the ratio of O_2 production and biomass synthesis, and $Y_{bio}^{N:C}$ (mol N mol C^{-1}) is the N:C ratio in total biomass. Also, we can stoichiometrically relate C fixation rate to the photosynthetic O_2 production rate P_{O2} (mol O_2 cell^{-1} s^{-1}):

$$P_{O2} = P_I \ Chl Y_{photo}^{O2:CH} \qquad \text{[eq. S12]}$$

where $Y_{photo}^{O2:CH}$ (mol O_2 mol C^{-1}) is the O_2/C ratio in the photosynthesis. Using these rates (P_I, λ, P_{Cstore}, Exc) in [eq. 5] ~ [eq. 7] and step forward these equations, we obtain values for the next time step. During the light period, N_{2fix}, P_{CO2}^{N2fix} and P_{CO2}^{RP} are assumed zero.

We assume that the intracellular O_2 rapidly equilibrates (as is reasonable for small cells). From the respiration rate R_{O2} and the O_2 production rate P_{O2} and [eq. 8] with a pseudo-steady state assumption, V_{O2} (mol O_2 cell^{-1} s^{-1}) is calculated as follows:

$$V_{O2} = R_{O2} - P_{O2}. \qquad \text{[eq. S13]}$$

This pseudo-steady state assumption is valid in this study since the amount of intracellular O_2 is significantly smaller than that of cellular C despite the magnitude of fluxes affecting these pools is similar. With [eq. 2], by assuming a spherical shape of the cells, we obtain the intracellular O_2 concentration $[O_2^{cell}]$ as follows:

$$[O_2^{cell}] = [O_2\ ] - \frac{V_{O2}}{4\pi r \kappa_{O2}}. \qquad \text{[eq. S14]}$$

Dark period. During the dark period, the rates of N_2 fixation and respiration are influenced by the amount of stored C, the electron and energy source for both N_2 fixation and respiration. The potential C storage decomposition rate (mol C cell^{-1} s^{-1}) is based on the availability of the C storage and represented by the following Michaelis-Menten form:

$$D_{Cs}^{potential} = -D_{Cs}^{max} \frac{C_S}{C_S\ + K_{Cs}^{dec}} \qquad \text{[eq. S15]}$$

where D_{Cs}^{max} (mol C cell^{-1} s^{-1}) is the maximum rate of C storage decomposition, and K_{Cs}^{dec} (mol C cell^{-1}) is the half saturation constant of the C storage for $D_{Cs}^{potential}$. This $D_{Cs}^{potential}$ imposes the limit of N_2 fixation and respiration (see below). In addition, respiration is limited by the potential O_2 uptake rate (mol O_2 cell^{-1} s^{-1}) and the enzymatically constrained respiratory potential. The potential O_2 uptake is obtained when the intracellular O_2 concentration is zero:

$$V_{O2}^{potential} = 4\pi r \kappa_{O2}[O_2\ ] \qquad \text{[eq. S16]}$$

The enzymatically constrained respiratory potential (mol O_2 cell^{-1} s^{-1}) is assumed to increase with time after the initiation of the dark period and is represented as follows:

$$R_{enzyme}^{potential} = (t_{dark})^{P1} C_{O2}^{potential} \qquad \text{[eq. S17]}$$

where t_{dark} (s) is the time passed since the initiation of the dark period, $P1$ (dimensionless) and $C_{O2}^{potential}$ (unit depends on $P1$) are the power factor and the respiratory coefficient for $R_{enzyme}^{potential}$. We have used this power factor in order to express the observed non-linear time dependence of respiration during the dark period (8). If $V_{O2}^{potential} > R_{enzyme}^{potential}$, the potential respiration rate $R_{O2}^{potential}$ (mol O_2 cell^{-1} s^{-1}) is equal to $R_{enzyme}^{potential}$, whereas $V_{O2}^{potential} \leq R_{enzyme}^{potential}$, it is equal to $V_{O2}^{potential}$ (mol O_2 cell^{-1} s^{-1}).

Once we obtain the potential respiration rate, the next step is to compute the potential N_2 fixation rate. The potential N_2 fixation rate (mol N cell^{-1} s^{-1}) is constrained by the availability of nitrogenase enzyme, as well as intracellular O_2:

$$N_{2fix}^{potential} = Fe_N\, C_{Fe}^{N2fix} \frac{[O_2^{cell}]_{cri} - [O_2^{cell}]}{[O_2^{cell}]_{cri}} \qquad \text{[eq. S18]}$$

where Fe_N (mol Fe cell^{-1}) is the amount of Fe in nitrogenase (see below), C_{Fe}^{N2fix} (mol N mol Fe^{-1} s^{-1}) is the N_2-fixing capacity per nitrogenase Fe, and $[O_2^{cell}]_{cri}$ (mol O_2 m^{-3}) is a critical O_2 concentration above which N_2 fixation cannot occur (thus, when $[O_2^{cell}] > [O_2^{cell}]_{cri}$, $N_{2fix}^{potential} = 0$).

We now can ask whether the cell is N or C limited: i.e. whether there is enough C stored to meet the demands of potential N_2 fixation. To do that, we first define the ideal C storage decomposition rate D_{Cs}^{ideal} (mol C cell^{-1} s^{-1}) based on those N_2 fixation and respiration rates:

$$D_{Cs}^{ideal} = \frac{N_{2fix}^{potential} Y_{N2fix}^{C:N}}{R_{H2}} + \frac{R_{O2}^{potential}}{Y_{non-synth}^{O2:CH}} \quad \text{[eq. S19]}$$

where $Y_{N2fix}^{C:N}$ (mol C mol N^{-1}) is the ratio of carbohydrate consumption (for electron donation for N_2 fixation) to N_2 fixation, R_{H2} (dimensionless) is a coefficient for electron recycling from hydrogen molecules, and $Y_{non-synth}^{O2:CH}$ (mol O_2 mol C^{-1}) is the ratio of O_2 consumption and carbohydrate consumption in non-synthesis respiration (2, 3). If $D_{Cs}^{ideal} < D_{Cs}^{potential}$, the decomposition rate of C storage D_{Cs} is controlled by D_{Cs}^{ideal} and $N_{2fix} = N_{2fix}^{potential}$. If $D_{Cs}^{ideal} > D_{Cs}^{potential}$, $D_{Cs}^{potential}$ controls the rate D_{Cs}, and the N_2 fixation rate is down regulated by $D_{Cs}^{potential}$:

$$N_{2fix} = \left(\frac{D_{Cs}^{potential} Y_{non-synth}^{O2:CH} - R_{O2}^{potential}}{Y_{N2fix}^{C:N} Y_{non-synth}^{O2:CH}} \right) R_{H2} \quad \text{[eq. S20]}$$

If $R_{O2}^{potential} > D_{Cs}^{potential}$, there is no N_2 fixation and the respiration rate equals $D_{Cs}^{potential}$.

Finally, we test if there is enough respiration for producing energy for N_2 fixation R_{N2fix} (mol O_2 cell^{-1} s^{-1}). The respiratory requirement is proportional to N_2 fixation:

$$R_{N2fix} = N_{2fix} Y_{N2fix}^{O2:N} \quad \text{[eq. S21]}$$

where $Y_{N2fix}^{O2:N}$ is a conversion factor (mol O_2 mol N^{-1}). When $R_{O2}^{potential} > R_{N2fix}$, $R_{O2} = R_{O2}^{potential}$, and otherwise, we impose $R_{O2} = R_{N2fix}$ as long as C store is available, which we have confirmed in this study. Under the pseudo-steady state of cellular O_2 and zero photosynthesis, from [eq. 8]:

$$V_{O2} = R_{O2} \quad \text{[eq. S22]}$$

And from [eq. S14], intracellular O_2 concentration is obtained. CO_2 production rate is computed as follows:

$$P_{CO2}^{N2fix} + P_{CO2}^{RP} = \frac{R_{O2}}{Y_{non-synth}^{O2:CH}} \quad \text{[eq. S23]}$$

Equating D_{Cs} to $-dC_S/dt$ and plugging these obtained values (N_{2fix}, R_{O2}, $P_{CO2}^{N2fix} + P_{CO2}^{RP}$) into [eq. 5] and [eq. 7], and applying a finite-difference method, we can compute the values for each time step. Here P_I, λ, *Exc* are assumed zero. When we turn off respiratory protection (thus $P_{CO2}^{RP} = 0$), $P_{CO2}^{N2fix} = R_{N2fix}/Y_{non-synth}^{O2:CH}$.

Computation of Fe metabolism

Light period. We have simulated the translocation of Fe among different cellular components as indicated in a proteomic study (9); photosystem Fe, buffer Fe, and nitrogenase Fe. Computing Fe flux and Fe content in each component is essential in predicting photosynthesis and N_2 fixation. The rate of Fe translocation is influenced by the amount of Fe in each location.

When $Chl^{ideal} > Chl$ there is translocation of Fe from buffer to the photosystem, whose flux F_B^P (mol Fe cell^{-1} s^{-1}) is represented as follows:

$$F_B^P = (Fe_P^{ideal} - Fe_P)R_B^P \frac{Fe_B}{Fe_B + K_{Fe}} \quad \text{[eq. S24]}$$

where Fe_P^{ideal} (mol Fe cell^{-1}) is the ideal Fe mass in the photosystem ($=Chl^{ideal}Y_{photo}^{Chl:Fe}$), Fe_P (mol Fe cell^{-1}) is the actual Fe mass in the photosystem, R_B^P (s^{-1}) is the photosystem production efficiency, Fe_B (mol Fe cell^{-1}) is the Fe mass in the buffer, and K_{Fe} (mol Fe cell^{-1}) is the half saturation constant of Fe metabolisms. We assume that the amount of the photosystem Fe per chlorophyll is constant so that Fe_P^{ideal} (mol Fe cell^{-1}) is obtained from Chl^{ideal} calculated in equation [eq. S3]. R_B^P is a function of time in the light period:

$$R_B^P = t_{light} C_B^P \quad \text{[eq. S25]}$$

where C_B^P (s^{-2}) is a constant value. This time function is to represent the observed gradual increases in Fe^P (9).

When $Chl_{ideal} \le Chl$, there is a flux of Fe from the photosystem to the buffer F_P^B (mol Fe cell^{-1} s^{-1}), represented as follows:

$$F_P^B = (Fe_P - Fe_P^{ideal}) R_P^B \frac{Fe_P}{Fe_P + K_{Fe}} \quad \text{[eq. S26]}$$

where R_P^B (s^{-1}) is the rate constant for F_P^B. In addition to the Fe transfer between the photosystem and the buffer, there can be a flux of Fe from nitrogenase to the buffer F_N^B (mol Fe cell^{-1} s^{-1}) if there is still some remaining Fe in nitrogenase (this flux tends to occur during the early light period):

$$F_N^B = Fe_N \, R_N^B \frac{Fe_N}{Fe_N + K_{Fe}} \quad \text{[eq. S27]}$$

where Fe_N (mol Fe cell^{-1}) is the amount of Fe in nitrogenase, R_N^B (s^{-1}) is the rate constant for F_N^B, and K_{Fe}^N (mol Fe cell^{-1}) is the nitrogenase Fe half saturation constant for the nitrogenase-buffer Fe transfer. Finally, F_B^N is assumed to be zero. With these fluxes, we predict the mass of Fe in each cell component. Fe_P, Fe_B, Fe_N and Chl ($= Fe_P Y_{photo}^{Chl:Fe}$) are computed based on the balances of the above Fe fluxes for each time step [eq. 9]~[eq. 11].

Dark period. During the early dark period, Fe atoms move from the photosystem to the buffer, and the buffer to nitrogenase. The flux of Fe from the photosystem to the buffer (mol Fe cell^{-1} s^{-1}) is based on the following equation:

$$F_P^B = (Fe_P - Fe_P^{min})R_P^B \frac{Fe_P}{Fe_P + K_{Fe}}$$ [eq. S28]

where Fe_P^{min} (mol Fe cell^{-1}) is the minimum cellular Fe in the photosystem and other variables are the same as in equation [eq. S26]. The flux of Fe from the buffer to nitrogenase (mol Fe cell^{-1} s^{-1}) is represented as follows in a similar form:

$$F_B^N = (Fe_N^{ideal} - Fe_N)R_B^N \frac{Fe_B}{Fe_B + K_{Fe}}$$ [eq. S29]

where Fe_N^{ideal} (mol Fe cell^{-1}) is the ideal Fe mass in the nitrogenase that fulfills the potential N_2 fixation rate based on C storage availability, R_B^N (s^{-1}) is the rate constant for nitrogenase production. R_B^N is influenced by the O_2 concentration, dark period time t_{dark} (s), and C storage:

$$R_B^N = C_B^N (t_{dark})^{P2} ([O_2^{cell}]_{cri}^{nitroge} - [O_2^{cell}]) \left(\frac{C_S}{C_S + K_{Cs}^{nitroge}}\right)^{P3}$$ [eq. S30]

where C_B^N (unit varies depending on *P2* and *P3*) is a constant term for R_B^N, *P2* and *P3* (dimensionless) are power factors, necessary to accurately represent repeatedly observed non-linear time dependence of N_2 fixation (8–10), $[O_2^{Cell}]_{cri}^{nitroge}$ (mol O_2 m^{-3}) is a critical O_2 concentration above which nitrogenase can be synthesized (thus, when $[O_2^{cell}] > [O_2^{cell}]_{cri}^{nitroge}$, $R_B^N = 0$), $K_{Cs}^{nitroge}$ (mol C cell^{-1}) is a half saturation constant of C_S for nitrogenase production.

Fe_N^{ideal} (mol Fe cell^{-1}) is computed based on the ideal N_2 fixation rate. First, we compute the ideal N_2 fixation rate based on the concentration of the N storage and C storage (mol N cell^{-1} s^{-1}):

$$N_{2fix}^{ideal-store} = \frac{N_S^{max} - N_S}{N_S^{max}} N_{2fix}^{max} \frac{C_S}{C_S + K_{Cs}^{N2fix}}$$ [eq. S31]

where N_S^{max} (mol N cell^{-1}) is the N storage capacity, N_{2fix}^{max} (mol N cell^{-1} s^{-1}) is the maximum possible N_2 fixation rate, and K_{Cs}^{N2fix} (mol C cell^{-1}) is the Half saturation constant of carbohydrate storage for N_2 fixation. Then, we compare this value to another ideal N_2 fixation rate (mol N cell^{-1}), which, this time, is based on the balance between maximum C storage decomposition and maximum respiration:

$$N_{2fix}^{ideal-balance} = \left(\frac{D_{Cs}^{max}}{Y_{N2fix}^{CH:N}} - \frac{V_{O2}^{potential}}{Y_{non-synth}^{O2:CH} Y_{N2fix}^{CH:N}}\right) R_{H2} \quad \text{[eq. S32]}$$

We then take the lower value between $N_{2fix}^{ideal-store}$ and $N_{2fix}^{ideal-balance}$ for the ideal N_2 fixation rate N_{2fix}^{ideal} (mol N cell^{-1} s^{-1}), based on which, we compute the ideal Fe amount in nitrogenase:

$$Fe_N^{ideal} = \frac{N_{2fix}^{ideal}}{C_{Fe}^{N2fix}} \quad \text{[eq. S33]}$$

Based on the obtained fluxes through the above computations, Fe_P , Fe_B and Fe_N (mol Fe cell^{-1}), (cellular Fe in photosystems, Fe-buffer, and nitrogenase, respectively) are computed for each time step. F_N^B is assumed to be zero, but the flux of Fe from nitrogenase to the buffer can occur with a negative value of F_B^N. In each time step, we compute these fluxes and use them to predict the mass of Fe (and *Chl* $= Fe_P Y_{photo}^{Chl:Fe}$) for each time step through finite-difference method with [eq. 9]~[eq. 11].

Late dark period. During the late dark period, experimental data indicate that the cells start decomposing nitrogenase to retrieve Fe in the buffer, preparing for photosynthesis (9). Also, during this period, the respiration rate gradually decreases as the N_2 fixation rate decreases despite the available C storage (8). In this model, we imposed linearly decreasing F_N^{ideal} and $R_{O2}^{potential}$ with time, reaching $F_N^{ideal} = 0$ and half of the $R_{O2}^{potential}$ at the initiation of the next light period.

Computing cellular C and N. We consider total cellular C (Q_C) and N (Q_N) quotas as a sum of two pools: inflexible biomass (C_B and N_B) and flexible storage:

$$Q_C = C_B + C_S \qquad \text{[eq. S34]}$$

$$Q_N = N_B + N_S. \qquad \text{[eq. S35]}$$

Based on these values, the N:C ratio of the total biomass is obtained:

$$Y_{bio-all}^{N:C} = \frac{Q_N}{Q_C}. \qquad \text{[eq. S36]}$$

Impact of cell size.

All the metabolic rates (except for N_{2fix}^{max}), maximum storage capacity and baseline biomass concentrations are assumed proportional to the cell size.

Modeling T dependence.

To evaluate the effect of temperature (T), we multiplied $f_T(T)$ [eq. 3] to P_I, $\lambda^{Chl-ideal}$, $P_{Cs}^{Chl-ideal}$, $R_{enzyme}^{potential}$, and/or $N_{2fix}^{potential}$, depending on the simulation. To make all the metabolisms (photosynthesis, respiration and N_2 fixation) T dependent, we multiply $f_T(T)$ to all of these. To make only one of these metabolisms T dependent, the factor is multiplied only to P_I, $\lambda^{Chl-ideal}$ and $P_{Cs}^{Chl-ideal}$ for photosynthesis, to $R_{enzyme}^{potential}$ for N_2 fixation or to $N_{2fix}^{potential}$ for respiration, respectively.

Parameterization

Tunable parameters. In order to represent the previous experimental data (8, 9) as well as the new data from our study, we have visually estimated 34 parameters (Table S1). In addition, we have set that the

late dark period starts 7 hours after the initiation of the dark period. To reduce the number of free parameters, we have applied same values for *P2* and *P3*, and K_{Cs}^{dec} and K_{Cs}^{N2fix}. For the cellular Fe quota, we have used a constant value of 4.87×10^6 molecules per cell based on the maximum value estimated in the proteomic study (9).

Non-tunable parameters. In order to constrain other parameters, we have referred to Inomura et al., (2017) (1) for the following parameters; $Y_{synth}^{O2:Bio}$, $Y_{non-synth}^{O2:CH}$, $Y_{N2fix}^{O2:N}$, and E (with energy transfer efficiency of 0.41 (11) and biomass of $C_5H_7O_2N_a$), where a = $5Y_{bio-all}^{N:C}$ (C:H:O as suggested (1)). We assume that 100% electron from hydrogen produced from N_2 fixation, which leads to R_{H2} = 4/3 (8). Also, based on the electron balance between N_2 fixation and glucose decomposition, $Y_{N2fix}^{C:N}$=1 (2, 3). We have set the reference temperature T_{ref} = 28 ºC (301.15 K) following the reference experiments (8, 9) as well as the laboratory experiments in this study.

References

1. **Rittmann BE, McCarty PL**. 2001. Environmental Biotechnology: Principles and Applications. McGraw-Hill: New York, NY.
2. **Inomura K, Bragg J, Follows MJ**. 2017. A quantitative analysis of the direct and indirect costs of nitrogen fixation: a model based on *Azotobacter vinelandii*. ISME J **11**:166–175.
3. **Inomura K, Bragg J, Riemann L, Follows MJ**. 2018. A quantitative model of nitrogen fixation in the presence of ammonium. PLoS ONE **13**:e0208282.
4. **Webb WL, Newton M, Starr D**. 1974. Carbon dioxide exchange of *Alnus rubra* A mathematical model. Oecologia **17**:281–291.
5. **Platt T, Gallegos CL, Harrison WG**. 1980. Photoinhibitioin of photosynthesis in natural assemblages of marine phytoplankton. J Mar Res **38**:687–701.
6. **Cullen JJ**. 1990. On models of growth and photosynthesis in phytoplankton. Deep Res **37**:667–683.
7. **Geider RJ, Macintyre HL, Kana TM**. 1998. A dynamic regulatory model of phytoplanktonic acclimation to light, nutrients, and temperature. Limnol Oceanogr **43**:679–694.
8. **Großkopf T, LaRoche J**. 2012. Direct and indirect costs of dinitrogen fixation in *Crocosphaera watsonii* WH8501 and possible implications for the nitrogen cycle. Front Microbiol **3**:doi: 10.3389/fmicb.2012.00236.
9. **Saito MA, Bertrand EM, Dutkiewicz S, Bulygin V V., Moran DM, Monteiro FM, Follows MJ, Valois FW, Waterbury JB**. 2011. Iron conservation by reduction of metalloenzyme inventories in the marine diazotroph *Crocosphaera watsonii*. Proc Natl Acad Sci **108**:2184–2189.
10. **Mohr W, Intermaggio MP, LaRoche J**. 2010. Diel rhythm of nitrogen and carbon metabolism in the unicellular, diazotrophic cyanobacterium *Crocosphaera watsonii* WH8501. Environ

Microbiol **12**:412–421.

11. **Xiao J, VanBriesen JM**. 2005. Expanded thermodynamic model for microbial true yield prediction. Biotechnol Bioeng **93**:110–121.
