# Supplementary figures and images for: Quantifying Oxygen Management and Temperature and Light Dependencies of Nitrogen Fixation by Crocosphaera watsonii
Source: mSphere. 2019 Dec 11;4(6):e00531-19. doi: 10.1128/mSphere.00531-19 (PMC6908418; doi:10.1128/mSphere.00531-19)

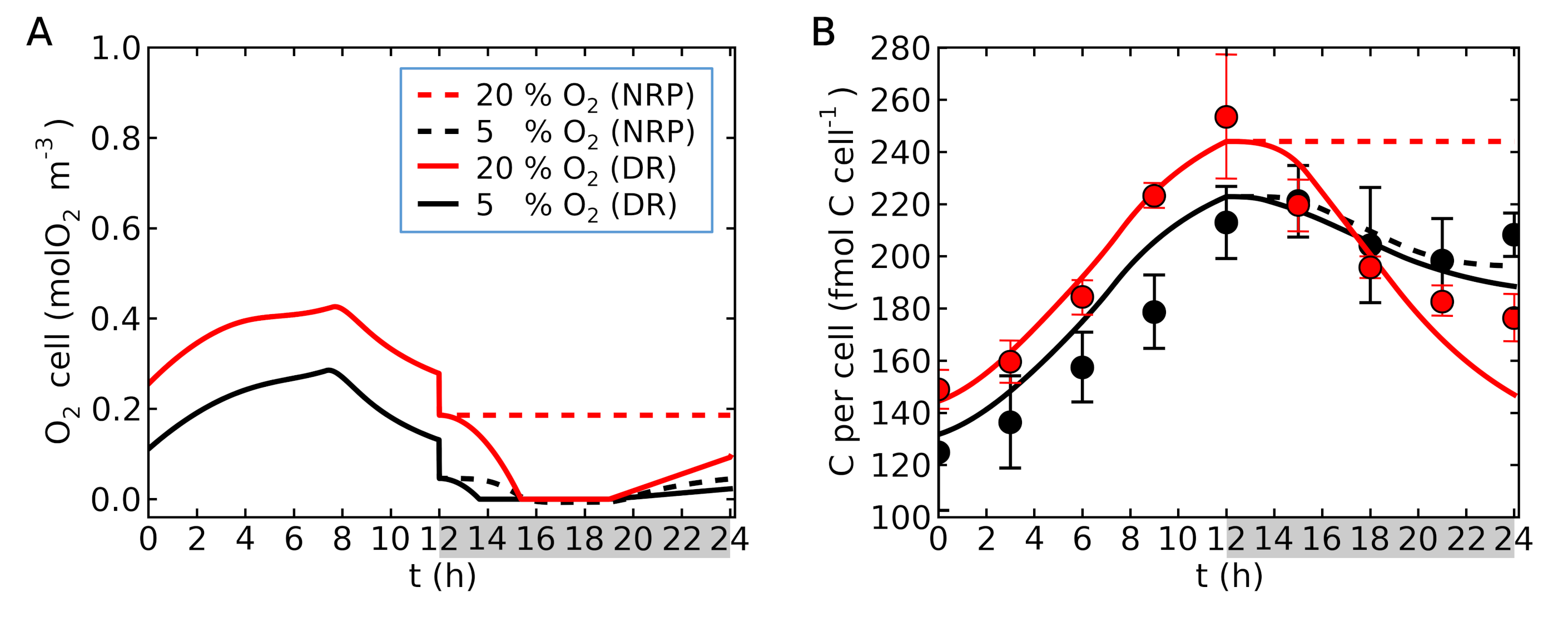

Supplement: FIG S1 [file mSphere.00531-19-sf001.tif]

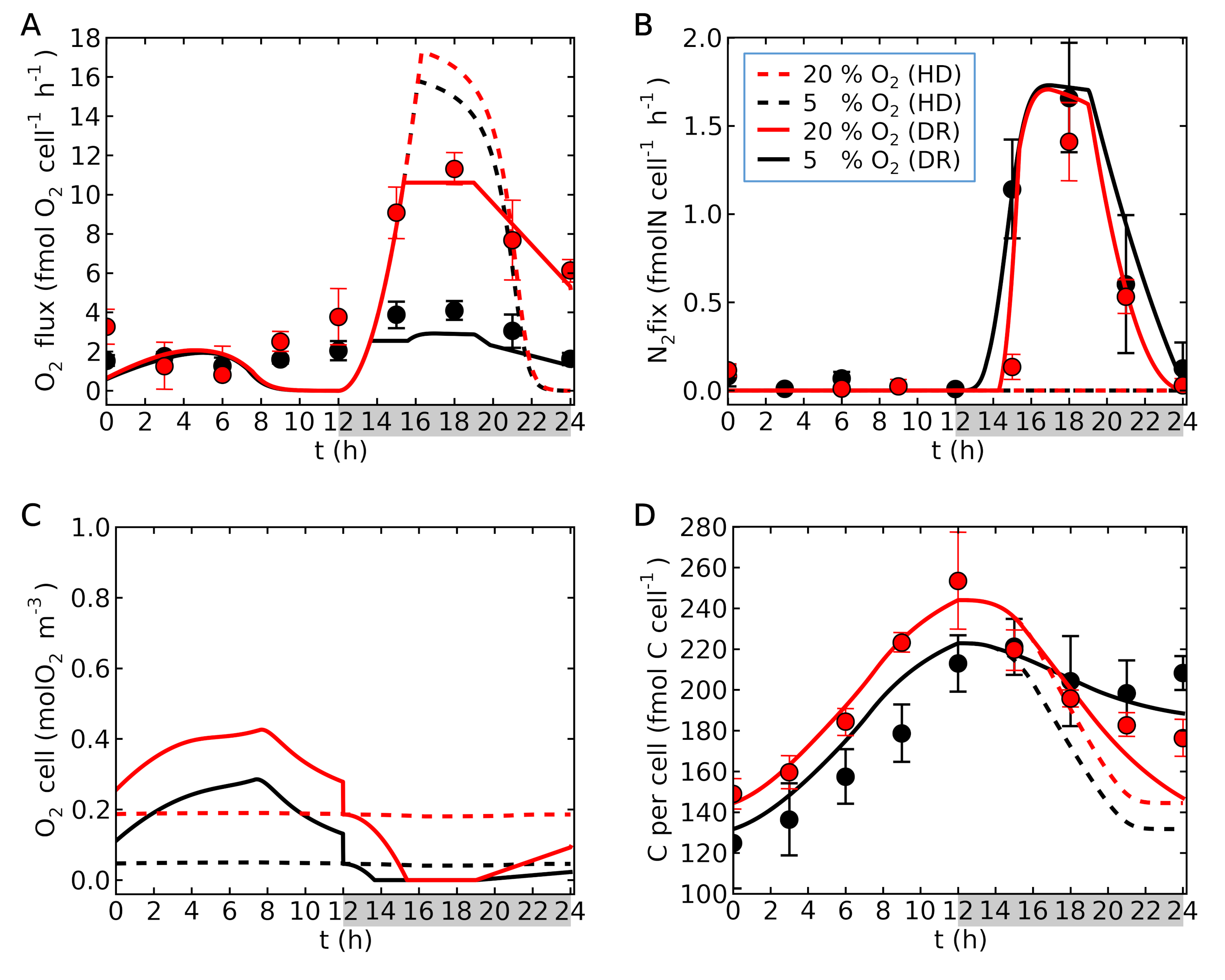

Supplement: FIG S2 [file mSphere.00531-19-sf002.tif]
