# Supplementary material for: Quantifying Oxygen Management and Temperature and Light Dependencies of Nitrogen Fixation by Crocosphaera watsonii
Source: mSphere. 2019 Dec 11;4(6):e00531-19. doi: 10.1128/mSphere.00531-19 (PMC6908418; doi:10.1128/mSphere.00531-19)
Supplement: TABLE S1 [file mSphere.00531-19-st001.pdf]

Parameter	Value	Unit
r	1.32×10^{-6}	m
ε_m	1.55×10^{-5}	dimensionless
$Y_{photo}^{Chl:Fe}$	1.91×10^{2}	mol C mol Fe^{-1}
λ^{max}	2.51×10^{-18}	mol C $cell^{-1}$ s^{-1}
C_S	0.00	mol C $cell^{-1}$
(initial value)		
N_S	2.21×10^{-15}	mol N $cell^{-1}$
(initial value)		
K_{Ns}	8.83×10^{-16}	mol N $cell^{-1}$
C_B	1.45×10^{-13}	mol C $cell^{-1}$
N_B	2.41×10^{-14}	mol N $cell^{-1}$
C_S^{max}	1.19×10^{-13}	mol C $cell^{-1}$
R_{Cs}	6.32×10^{-5}	s^{-1}
P_I^{max}	7.28×10^{-3}	s^{-1}
A_I	3.00×10^{-3}	μmol^{-1} m^2 s
B_I	6.46×10^{-1}	dimensionless
C_I	6.90×10^{-3}	μmol^{-1} m^2 s
D_I	7.76×10^{0}	dimensionless
A_T	8.00×10^{3}	Dimensionless

D_{Cs}^{max}	5.21×10^-18	mol C cell^{-1} s^{-1}
K_{Cs}^{Dec}	6.30×10^-15	mol C cell^{-1}
$P1$	2.00×10^0	dimensionless
$C_{O2}^{potential}$	2.00×10^-26	unit depends on *P1*
C_{Fe}^{N2fix}	1.00×10^-1	mol N mol Fe^{-1} s^{-1}
$[O_2^{cell}]_{cri}$	1.00×10^-1	mol O_2 m^{-3}
C_B^P	2.40×10^-1	s^{-1}
K_{Fe}	4.53×10^-19	mol Fe cell^{-1}
Fe_P^{min}	2.54×10^-18	mol Fe cell^{-1}
R_P^B	5.00×10^-4	s^{-1}
R_N^B	3.33×10^-3	s^{-1}
C_B^N	2.39×10^-15	unit depends on *P2*
$P2$	3.00×10^0	dimensionless
$[O_2^{cell}]_{cri}^{nitroge}$	3.26×10^-1	mol O_2 m^{-3}
$K_{Cs}^{nitroge}$	3.15×10^-14	mol C cell^{-1}
N_S^{max}	4.59×10^-13	mol N cell^{-1}
N_{2fix}^{max}	5.21×10^-19	mol N cell^{-1} s^{-1}

The parameters are for 20% O_2 environment. For 5% O_2 environment, we have used $r = 1.28 \times 10^{-6}$ (m) and other per-cell values except for N_{2fix}^{max} are proportionally adjusted based on the volume ($V = 4/3\pi r^3$) difference between the two O_2 cases. ε_m is a diffusivity of cellular membrane relative to water, which influences κ_{O2} (1).

Reference

1. **Inomura K, Bragg J, Follows MJ**. 2017. A quantitative analysis of the direct and indirect costs of nitrogen fixation: a model based on *Azotobacter vinelandii*. ISME J **11**:166–175.
