# Supplementary material for: Quantifying Oxygen Management and Temperature and Light Dependencies of Nitrogen Fixation by Crocosphaera watsonii
Source: mSphere. 2019 Dec 11;4(6):e00531-19. doi: 10.1128/mSphere.00531-19 (PMC6908418; doi:10.1128/mSphere.00531-19)
Supplement: TABLE S2 [file mSphere.00531-19-st002.pdf]

Parameter	Definition	Unit
N_{2fix}	N_2 fixation rate	mol N $cell^{-1}$ s^{-1}
$[O_2^{cell}]$	Cellular O_2 concentration	mol O_2 m^{-3}
$[O_2\ ]$	Environmental O_2 concentration	mol O_2 m^{-3}
r	Cell radius	m
γ_{net}	Net respiration rate	mol O_2 m^{-3} s^{-1}
κ_{O_2}	Effective O2 diffusion coefficient	m^2 s^{-1}
T	Temperature	K
T_{ref}	Reference temperature	K
A_T	Constant factor for T influence	Dimensionless
I	Light intensity	µmol m^{-2} s^{-1}
A_I	Power coefficient for photosynthesis	$µmol^{-1}$ m^2 s
C_S	C storage per cell	mol C mol $cell^{-1}$
t	Time	s
P_I	Photosynthesis rate per chlorophyll	s^{-1}
Chl	Chlorophyll per cell	mol C $cell^{-1}$
λ	Biomass production rate	mol C $cell^{-1}$ s^{-1}
E	CO_2 production : Biomass production	dimensionless
P_{CO2}^{N2fix}	CO_2 production due to electron donation to and respiratory energy production for N_2 fixation	mol C $cell^{-1}$ s^{-1}
P_{CO2}^{RP}	CO_2 production due to respiratory protection	mol C $cell^{-1}$ s^{-1}
Exc	Rate of carbohydrate excretion	mol C $cell^{-1}$ s^{-1}

X	Number density of cells	cell m^{-3}
Q_C	Cellular C quota	mol C $cell^{-1}$
Q_N	Cellular N quota	mol N $cell^{-1}$
N_S	N storage per cell	mol N mol $cell^{-1}$
$Y_{bio-all}^{N:C}$	N : C in biomass with nutrient storage	mol N mol C^{-1}
O_2	O_2 per cell	mol O_2 $cell^{-1}$
P_{O2}	O_2 production rate	mol O_2 $cell^{-1}$ s^{-1}
R_{O2}	Respiration rate	mol O_2 $cell^{-1}$ s^{-1}
V_{O2}	O_2 diffusion into the cell	mol O_2 $cell^{-1}$ s^{-1}
Fe_P	Fe in the photosystem per cell	mol Fe $cell^{-1}$
F_B^P	Translocation of Fe from the buffer to the photosystem	mol Fe $cell^{-1}$ s^{-1}
F_P^B	Translocation of Fe from the photosystem to the buffer	mol Fe $cell^{-1}$ s^{-1}
Fe_B	Fe in the buffer per cell	mol Fe $cell^{-1}$
F_B^N	Translocation of Fe from the buffer to nitrogenase	mol Fe $cell^{-1}$ s^{-1}
F_N^B	Translocation of Fe from nitrogenase to the buffer	mol Fe $cell^{-1}$ s^{-1}
Fe_N	Fe in nitrogenase per cell	mol Fe $cell^{-1}$
$Y_{photo}^{Chl:Fe}$	Chlorophyll to Fe ratio	mol C mol Fe^{-1}
$\lambda^{Chl\text{-}ideal}$	Potential biomass production rate under the ideal amount of chlorophyll	mol C $cell^{-1}$ s^{-1}

λ^{max}	Maximum biomass production rate	mol C cell^{-1} s^{-1}
K_{Ns}	Half-saturation constant of N storage for biomass production	mol N cell^{-1}
$Y_{bio}^{N:C}$	N : C in biomass without nutrient storage	mol N mol C^{-1}
$P_{Cs}^{Chl-ideal}$	Potential C storage production rate under the ideal amount of chlorophyll	mol C cell^{-1} s^{-1}
C_S^{max}	Maximum C storage per cell	C mol cell^{-1}
R_{Cs}	Rate constant for the production of C storage	s^{-1}
R_{bio}	Respiration for biosynthesis	mol O_2 cell^{-1} s^{-1}
Chl^{ideal}	Chlorophyll per cell	mol C cell^{-1}
P_I^{max}	Maximum photosynthesis rate per chlorophyll	s^{-1}
M_{Chl}	Chl : Chl_{ideal}	dimensionless
$Y_{synth}^{O2:bio}$	O_2 consumption : Biomass production in biosynthesis	mol O_2 mol C^{-1}
$Y_{photo}^{O2:CH}$	O_2 : Carbohydrate production in photosynthesis	mol O_2 mol C^{-1}
$D_{Cs}^{potential}$	Potential rate of carbohydrate storage decomposition based on the storage size	mol C cell^{-1} s^{-1}
D_{Cs}^{max}	Maximum rate of carbohydrate storage decomposition	mol C cell^{-1} s^{-1}
K_{Cs}^{dec}	Half saturation constant of the C storage for $D_{Cs}^{potential}$	mol C cell^{-1}
$V_{O2}^{potential}$	Potential O_2 uptake rate by diffusion	mol O_2 cell^{-1} s^{-1}

$R_{enzyme}^{potential}$	Enzymatically constrained respiratory potential	mol O_2 cell^{-1} s^{-1}
t_{dark}	Time passed since the initiation of the dark period	s
$P1$	Power factor for $R_{enzyme}^{potential}$	dimensionless
$C_{O2}^{potential}$	Respiratory coefficient for $R_{enzyme}^{potential}$	unit depends on $P1$
$R_{O2}^{potential}$	Potential respiration rate	mol O_2 cell^{-1} s^{-1}
$N_{2fix}^{potential}$	Potential rate of N_2 fixation	mol N cell^{-1} s^{-1}
C_{Fe}^{N2fix}	N_2-fixing capacity per nitrogenase Fe	mol N mol Fe^{-1} s^{-1}
$[O_2^{cell}]_{cri}$	Critical O_2 concentration above which N_2 fixation cannot occur	mol O_2 m^{-3}
D_{Cs}^{ideal}	Ideal rate of C storage decomposition	mol C cell^{-1} s^{-1}
$Y_{N2fix}^{C:N}$	Carbohydrate consumption : N_2 fixation	mol C mol N^{-1}
R_{H2}	Coefficient for electron recycling from hydrogen molecules	(dimensionless)
$Y_{non-synth}^{O2:CH}$	O_2 consumption : Carbohydrate consumption in non-synthesis respiration	mol O_2 mol C^{-1}
R_{N2fix}	Respiration for providing energy for N_2 fixation	mol O_2 cell^{-1} s^{-1}
$Y_{N2fix}^{O2:N}$	R_{N2fix} : N_{2fix}	mol O_2 mol N^{-1}
D_{Cs}	Rate of carbohydrate storage decomposition	mol C cell^{-1} s^{-1}
Fe_P^{ideal}	Ideal Fe mass in the photosystem	mol Fe cell^{-1}
R_B^P	Photosystem production efficiency	s^{-1}

t_{light}	Time passed since the initiation of the light period	s
C_B^P	Coefficient for R_B^P	s^{-2}
K_{Fe}	Half saturation constant of Fe metabolisms	mol Fe $cell^{-1}$
R_P^B	Rate constant for F_P^B	s^{-1}
R_N^B	Rate constant for F_N^B	s^{-1}
Fe_P^{min}	Minimum Fe amount in the photosystem	mol Fe $cell^{-1}$
Fe_N^{ideal}	Ideal Fe mass in nitrogenase	mol Fe $cell^{-1}$
R_B^N	Rate constant for F_B^N	s^{-1}
C_B^N	constant term for	unit depends on *P2*
P2	power factor for $R_{Buffer-Fe}^{Nitroge-Fe}$	dimensionless
$[O_2^{cell}]_{cri}^{nitroge}$	Critical O_2 concentration below which nitrogenase can be synthesized	mol O_2 m^{-3}
$K_{Cs}^{nitroge}$	Half saturation constant of C_S for nitrogenase production	mol C $cell^{-1}$
P3	Power term for carbohydrate-storage influence on nitrogenase synthesis	dimensionless
$N_{2fix}^{ideal-store}$	Ideal N_2 fixation rate based on the concentration of the N and C storage	mol N $cell^{-1}$ s^{-1}
N_S^{max}	Maximum N storage	mol N $cell^{-1}$
N_{2fix}^{max}	Maximum possible N_2 fixation rate	mol N $cell^{-1}$ s^{-1}

K_{Cs}^{N2fix}	Half saturation constant of carbohydrate storage for N_2 fixation	mol C $cell^{-1}$
$N_{2fix}^{ideal-balance}$	N fixation rate based on the balance between maximum C storage decomposition and maximum respiration	mol N $cell^{-1}$ s^{-1}
N_{2fix}^{ideal}	Ideal N_2 fixation rate	mol N $cell^{-1}$ s^{-1}
ε_m	Relative diffusivity of the cell membrane	dimensionless
